# Co-delivery of rhBMP-2 and zoledronic acid using calcium sulfate/hydroxyapatite carrier as a bioactive bone substitute to enhance and accelerate spinal fusion

**DOI:** 10.1016/j.bioactmat.2024.02.034

**Published:** 2024-03-06

**Authors:** Xinggui Tian, Corina Vater, Deepak Bushan Raina, Lisa Findeisen, Lucas-Maximilian Matuszewski, Magnus Tägil, Lars Lidgren, Anja Winkler, Robert Gottwald, Niels Modler, Klaus-Dieter Schaser, Alexander C. Disch, Stefan Zwingenberger

**Affiliations:** a University Center of Orthopaedic, Trauma and Plastic Surgery, University Hospital Carl Gustav Carus at TUD Dresden University of Technology, 01307, Dresden, Germany; b Center for Translational Bone, Joint and Soft Tissue Research, University Hospital Carl Gustav Carus at TUD Dresden University of Technology, 01307, Dresden, Germany; c Lund University, Faculty of Medicine, Department of Clinical Sciences Lund, Orthopaedics, Lund, 22185, Sweden; d Institute of Lightweight Engineering and Polymer Technology at TUD Dresden University of Technology, 01062, Dresden, Germany

**Keywords:** Calcium sulfate/hydroxyapatite, Bone morphogenetic protein 2, Bisphosphonate, Spinal fusion, Bone substitute

## Abstract

Recombinant human bone morphogenetic protein-2 (rhBMP-2) has been FDA-approved for lumbar fusion, but supraphysiologic initial burst release due to suboptimal carrier and late excess bone resorption caused by osteoclast activation have limited its clinical usage. One strategy to mitigate the pro-osteoclast side effect of rhBMP-2 is to give systemic bisphosphonates, but it presents challenges with systemic side effects and low local bioavailability. The aim of this *in vivo* study was to analyze if posterolateral spinal fusion (PLF) could be improved by utilizing a calcium sulfate/hydroxyapatite (CaS/HA) carrier co-delivering rhBMP-2 and zoledronic acid (ZA). Six groups were allocated (CaS/HA, CaS/HA + BMP-2, CaS/HA + systemic ZA, CaS/HA + local ZA, CaS/HA + BMP-2 + systemic ZA, and CaS/HA + BMP-2 + local ZA). 10-week-old male Wistar rats, were randomly assigned to undergo L4-L5 PLF with implantation of group-dependent scaffolds. At 3 and 6 weeks, the animals were euthanized for radiography, μCT, histological staining, or biomechanical testing to evaluate spinal fusion. The results demonstrated that the CaS/HA biomaterial alone or in combination with local or systemic ZA didn't support PLF. However, the delivery of rhBMP-2 significantly promoted PLF. Combining systemic ZA with BMP-2 didn't enhance spinal fusion. Notably, the co-delivery of rhBMP-2 and ZA using the CaS/HA carrier significantly enhanced and accelerated PLF, without inhibiting systemic bone turnover, and potentially reduced the dose of rhBMP-2. Together, the treatment regimen of CaS/HA biomaterial co-delivering rhBMP-2 and ZA could potentially be a safe and cost-effective off-the-shelf bioactive bone substitute to enhance spinal fusion.

## Highlights

• Calcium sulfate/hydroxyapatite (CaS/HA) biomaterial is an optimized delivery carrier for rhBMP-2 to promote spinal fusion.
• Local ZA application based on CaS/HA has highly targeted biological effects, avoidingconcerns about systemic side effects.
• CaS/HA co-delivery of FDA-approved rhBMP-2 and ZA, synergistically enhances and accelerates spinal fusion.
• CaS/HA-controlled ZA co-delivery may reduce rhBMP-2 dosage, improve its safety and be cost-effective.
• CaS/HA, rhBMP-2 and ZA) have been clinically approved, simplifying the clinical translation of this treatment regimen.

## Graphical abstract

**Figure:**
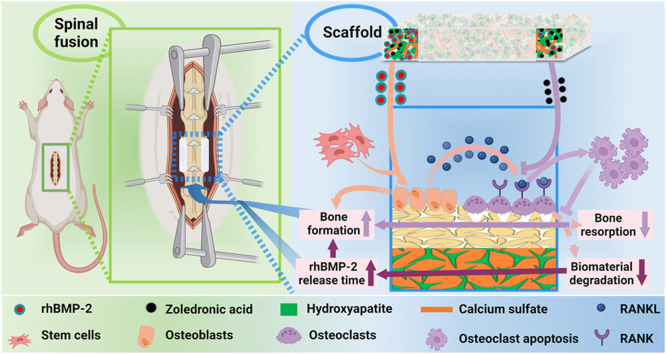

## 1 Introduction

Spinal fusion is a standard treatment for various spinal disorders, including trauma, deformity, degenerative disorders, tumors and infections, with more than 400,000 surgical procedures performed annually in the USA alone [1,2]. The total number of spinal fusion procedures has been on the rise over the past few decades globally, mainly due to improvements in surgical techniques, better implants and expansion of surgical indications [1]. Posterolateral spinal fusion (PLF) is a common surgical procedure to treat spinal instability [[3], [4], [5]]. The permanent stability of the procedure relies on bony fusion achieved by bone grafting between the transverse processes [[5], [6], [7]]. A recent review reported that PLF has the lowest fusion success rate compared to all other spinal fusion techniques [7]. The PLF failure rate ranges between 10 % and 55 %, impacting the final functional outcome [8]. Autologous bone grafting, usually taken from the iliac crest, is considered as the “gold standard” for successful fusion in PLF [3]. But the availability of autologous bone is limited and the PLF procedure requires large volumes of bone graft [5]. Furthermore, harvesting autologous bone is associated with donor site-related complications, including pain, infection and sensory abnormalities [4]. Allografts are attractive alternatives to autografts, but they carry potential risks of disease transmission and immunogenicity [5]. Therefore, the development of an off-the-shelf, bioactive, synthetic bone graft substitute with high bone formation capacity is a promising treatment modality to accelerate fusion and reduce failures in PLF procedures.

Although biomaterials can provide an osteoconductive template for bone formation, they often lack the osteoinductive properties necessary for formation of large volumes of bone. In 2002, the recombinant human bone morphogenetic protein 2 (rhBMP-2) was approved by the Food and Drug Administration (FDA) for clinical use and is currently the primary clinically available alternative to autologous bone grafts [3,9,10]. In the field of spine surgery, rhBMP-2 is FDA-approved for single-stage anterior lumbar interbody fusion (ALIF) within a specific interbody cage [11,12] and its usage has increased over the past few decades [9]. In fact, off-label use of rhBMP-2 is also increasing in other spinal procedures as a clinical alternative to autograft bone, including posterior lumbar interbody fusion (PLIF), transforaminal lumbar interbody fusion (TLIF), and PLF [3,9]. In the last decade, multiple meta-analyses reported that rhBMP-2 has superior spinal fusion rates compared with autologous iliac crest bone in lumbar spinal fusion [13,14], with similar clinical results in PLF [8]. However, recent reviews found no clear advantage of using rhBMP-2 compared to iliac crest bone graft and side effects associated with rhBMP-2 were observed over the past decade, including inflammation, radiculopathy, ectopic bone formation, osteolysis, genitourinary events, and wound complications, which all limit its clinical application in spinal surgery [3,9,10]. Numerous reports indicate that supra-physiological amounts of rhBMP-2 are carrier-related, with sub-optimal absorbable collagen sponges (ACS) leading to burst release as the main cause of the adverse events [3,9,10]. In addition, secondary osteolysis due to rhBMP-2-activated osteoclasts as well as osteoblasts via the RANKL-RANK (osteoblast-preosteoclast) interaction [15], may lead to disc space collapse, osteolytic cystic lesions, implant displacement, loosening, or subsidence [9]. The side effects of rhBMP-2 may be one of the main reasons for the sharp decline in BMP usage in spinal fusion techniques in recent years [16].

The pharmacokinetics of drug release from the carrier are critical in terms of safety and success in delivering osteoinductive proteins for spinal fusion, because burst release may lead to bone shell formation and inflammation [17]. One strategy to eliminate osteoclast-related side effects is to combine rhBMP-2 with bisphosphonates, which could reduce rhBMP-2-induced bone resorption and increase the net new bone formation [9]. Zoledronic acid (ZA), a potent third generation bisphosphonate, induces apoptosis of osteoclasts [18,19]. Over the last decade, substantial pre-clinical evidence has been published regarding the balance between anabolism and catabolism (anabolic/anticatabolic paradigm) in bone formation. To date, there is only one *in vivo* study showing that rhBMP-2 in combination with systemic ZA administration significantly promoted spinal fusion [18]. Bisphosphonates bind to hydroxyapatite and are commonly administered systemically as an intravenous infusion to treat osteoporosis or bone metastases [19]. Initial bisphosphonate-associated adverse events that occur after intravenous therapy include an acute-phase response and hypocalcaemia [20]. Notably, long-term use of bisphosphonates has further been associated with pathological atypical fractures, due to impaired bone remodeling [[19], [20], [21]]. Systemic administration gives low drug concentration at the target site [22] whereas *in situ* administration of bisphosphonate via a local drug delivery system has shown to be effective at the desired target site [22] as well as circumvent the potential clinical side effects. However, in light of current literature, there are no studies on the local co-delivery of rhBMP-2 and ZA for spinal fusion.

Although the field of bone tissue engineering is growing rapidly, the challenge to develop an optimal controlled-release carrier acting as an efficient scaffold for co-delivery of rhBMP-2 and ZA has not been solved yet. As mentioned above, the ACS carrier co-packaged with rhBMP-2 in the Medtronic® product is considered to be suboptimal [3,9,10]. At present, BMP carriers include natural polymers, synthetic polymers, inorganic materials, and composites of the above-mentioned materials [23]. Most of them are used mainly in preclinical research and cannot be directly translated into clinical applications. The resorbable calcium sulfate/hydroxyapatite (CaS/HA; Cerament™ Bone Void Filler) biomaterial used in this study has been approved for human use by regulatory agencies in Europe and North America for the purpose of filling bone voids [24,25]. CaS/HA provides a simple platform for surgical application of biomaterials. The pre-packed composition consists of a premixed powder with a weight ratio of 60 (wt%) CaS and 40 wt% HA and an aqueous non-ionic radiocontrast agent. After mixing both components, the biomaterial is injectable and sets *in situ* to form a solid, but resorbable scaffold [26]. As per the manufacturer [27], the mechanical properties i.e. the compressive strength of the material in dry conditions ranges between 65 and 75 MPa, which is stronger than cancellous bone while the mechanical properties of the material in wet conditions ranges between 10 and 12 MPa. Our group has previously reported on successful co-delivery of rhBMP-2 and ZA using the CaS/HA biomaterial in an ectopic muscle pouch model [15]. The CaS/HA scaffold provided a controlled and long-term delivery of rhBMP-2 and ZA and more new bone was formed in comparison with rhBMP-2 alone. Surprisingly, a recent study also showed an excellent and cumulative effect on bone regeneration when rhBMP-2 and ZA are combined by the CaS/HA biomaterial in a rat femoral critical-size bone defect model [26]. The aim of this study therefore was to locally co-deliver FDA-approved bone-active drugs i.e. rhBMP-2 and ZA using a well-tested CaS/HA biomaterial to treat spinal fusion. Based on the existing results from previous studies [26,28], it was hypothesized that CaS/HA-mediated controlled co-delivery of rhBMP-2 and ZA could enhance spinal fusion and act as an off-the-shelf substitute to autologous bone transplantation.

## 2 Results

### 2.1 Study design

**Fig. 1:**
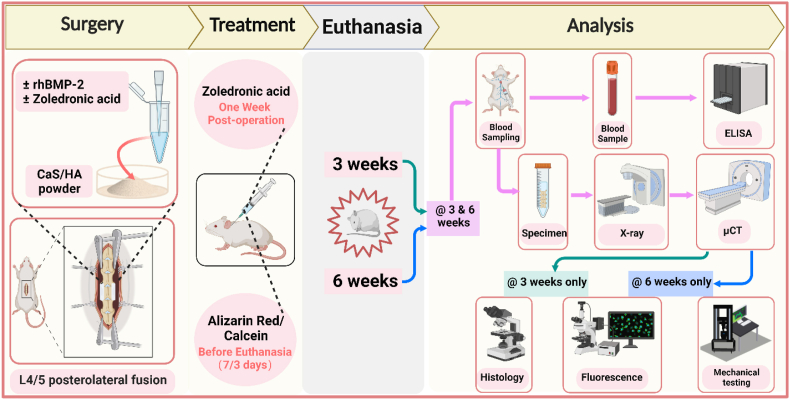
Schematic diagram of the methodology, timeline and evaluation techniques for this experimental study. Postoperative zoledronic acid therapy was only used in the systemic ZA administration (CaS/HA + ZA-s, CaS/HA + BMP + ZA-s) groups, and Alizarin Red and Calcein staining was only administrated in animals requiring fluorescent labeling *in vivo*. The histology indicates special histological staining. The green, blue, and purple arrows in analysis section indicate experimental analyses performed at 3 weeks only, 6 weeks only, and at 3 & 6 weeks, respectively. This figure was made on BioRender.com.

Based on the defined treatment with implant types, 6 groups were set up in this study: [1] CaS/HA, [2] CaS/HA + rhBMP-2 (CaS/HA + BMP), [3] CaS/HA + systemic ZA (CaS/HA + ZA-s), [4] CaS/HA + local ZA (CaS/HA + ZA-l), [5] CaS/HA + rhBMP-2 + systemic ZA (CaS/HA + BMP + ZA-s), [6] CaS/HA + rhBMP-2 + local ZA (CaS/HA + BMP + ZA-l). A PLF surgery at lumbar 4–5 bilaterally in 132, 10-week-old Wistar rats was performed by implanting group-dependent (Group 1-6) scaffolds. After 3 weeks, 12 animals per group and after 6 weeks 10 animals per group were euthanized to perform X-ray testing, micro‐computed tomography (μCT) scans, blood analysis and histological analysis, or biomechanical testing. The schematic diagram of methodology, timeline and evaluation techniques is given in Fig. 1. In the CaS/HA group, two rats (one from 3 weeks and one from 6 weeks) died for unknown reasons during observation. Additionally, one rat from both the CaS/HA and CaS/HA + BMP groups at 6 weeks were excluded due to surgical site infection. Furthermore, one sample each from CaS/HA + ZA-s and CaS/HA + BMP + ZA-s at 6 weeks were excluded from μCT analysis due to CT scan failure. During biomechanical testing at 6 weeks, one sample from CaS/HA + ZA-s group and one from CaS/HA + ZA-l group were excluded due to technical issues. Moreover, at the 3-week time point, two samples from each group intended for microarray analysis could not be included due to RNA extraction challenges. A detailed list of sample size for each experimental time point and technique is presented in Table S1.

### 2.2 Radiographic imaging: CaS/HA scaffold-mediated delivery of rhBMP-2 promotes PLF

**Fig. 2:**
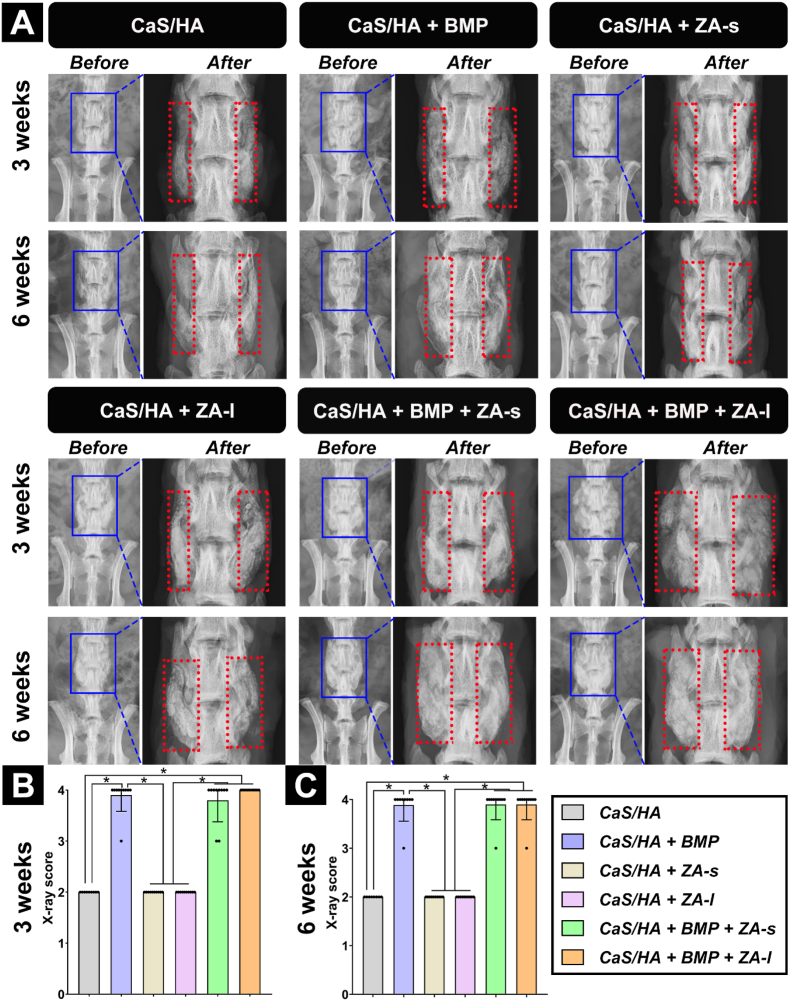
(**A**) Representative X-ray images before and after explanation at 3 and 6 weeks and X-ray spinal fusion score according to Curylo et al. score classification [29] at (**B**) 3 weeks and (**C**) 6 weeks. The blue and the red rectangle area within the images indicate the lumbar surgical site and the implanted scaffold region, respectively. “Before” and “After” represent the X-ray detection before and after transplantation, respectively. Data are presented as means ± SD. *p < 0.05.

Scaffold disintegration and rejection were observed in one rat each of the CaS/HA and CaS/HA + BMP group at 6 weeks, indicating infection of the surgical site combined with the wound conditions. Newly formed bone could be observed within the scaffold not beyond the scaffold area and no heterotopic ossification was observed from the before and after transplantation images. In the rhBMP-2-free groups (CaS/HA, CaS/HA + ZA-s, CaS/HA + ZA-l), no transverse process bridging was observed. In groups with scaffolds containing ZA (CaS/HA + ZA-l, CaS/HA + BMP + ZA-l), the scaffold material fractured at its position between the transverse processes in multiple segments. In groups containing rhBMP-2 (CaS/HA + BMP, CaS/HA + BMP + ZA-s, CaS/HA + BMP + ZA-l), a bony bridge formed to connect the adjacent transverse processes. According to the radiograph spinal fusion score classification defined by Curylo et al. [29], the groups containing rhBMP-2 had significantly higher scores than the rhBMP-2-free groups at both time points. Thereby, bilateral bone bridging was observed in 90 % of the animals and unilateral bone bridging in 10 %. No significant difference was observed among the rhBMP-2-free groups, and also in between the groups containing rhBMP-2 (Fig. 2, Fig. S1).

### 2.3 Micro-CT: CaS/HA-mediated co-delivery of rhBMP-2 and ZA enhances and accelerates PLF

**Fig. 3:**
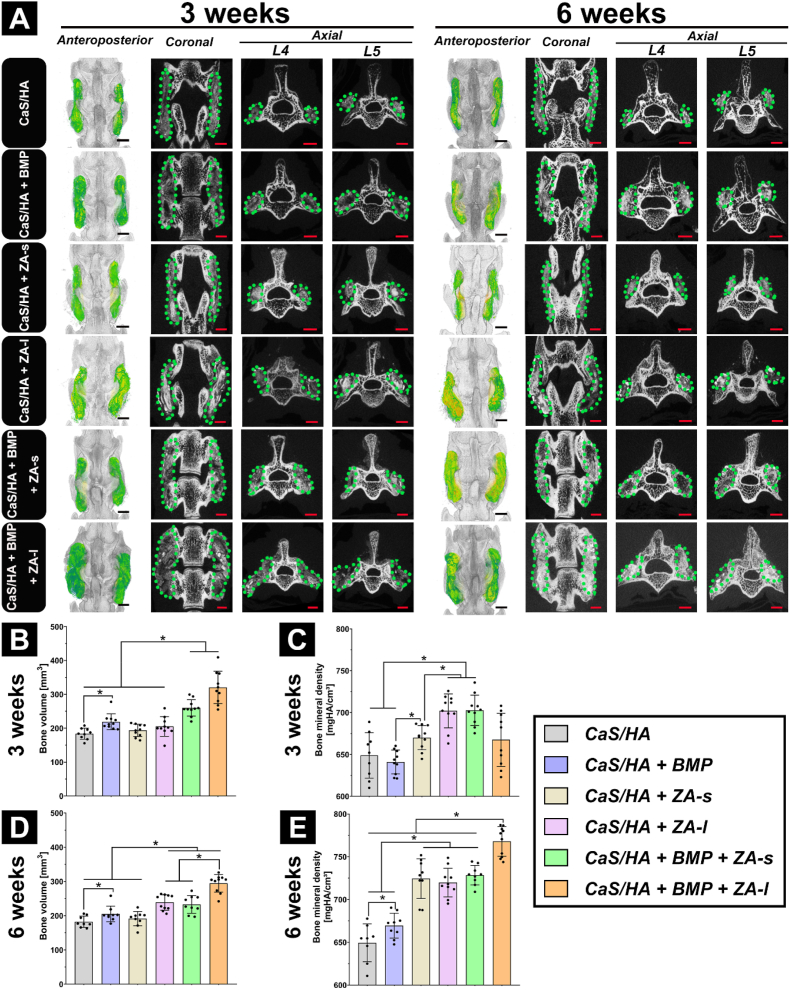
μCT–based evaluation of spinal fusion after treatment. (**A**) Representative 3D (anteroposterior view) reconstruction images (left) and corresponding 2D (coronal and axial) slices (right) obtained at 3 weeks and 6 weeks. (**B–C**) Bone volume and bone mineral density at 3 weeks and (**D-E**) at 6 weeks quantified in the lumbar fusion area (ROI) in all treatment groups. The green rendered areas in the reconstruction images represent the implanted scaffold and new bone formation zones. Data are presented as means ± SD. *p < 0.05, scale bars = 2 mm.

In rhBMP-2-free groups, the newly formed bone was not visible within the scaffold and the boundary between the scaffold and the native bone was clear. Newly formed bone was observed only at the base of both decorticated regions at both time points. In the groups containing rhBMP-2, new bone growth could be observed along the length of the entire scaffold resulting in PLF. At both time points, more new bone was generated in the CaS/HA + BMP + ZA-l group than in any of the other groups. In the CaS/HA + BMP and CaS/HA + BMP + ZA-s groups, multiple cystic lesions were visible within the new bone formation zones, but no cystic lesion was found in the CaS/HA + BMP + ZA-l group. Locally loaded ZA slowed down the degradation of the scaffolds, as evidenced by a significantly higher density of CaS/HA + ZA-l and CaS/HA + BMP + ZA-l samples compared to CaS/HA and CaS/HA + ZA-s, and CaS/HA + BMP and CaS/HA + BMP + ZA-s, respectively. The bone volume (BV) in the region of interest (ROI) showed that CaS/HA + BMP was higher than CaS/HA, and the BV created by CaS/HA + BMP + ZA-s and CaS/HA + BMP + ZA-l was higher than that of the other groups at 3 weeks, indicating that rhBMP-2 was optimally released from the CaS/HA carrier and promoted new bone formation. This effect was enhanced in combination with ZA application. The bone mineral density (BMD) quantification at 3 weeks showed CaS/HA + BMP and CaS/HA had the lowest BMD among all groups, while CaS/HA + BMP + ZA-l was between CaS/HA + BMP + ZA-s and CaS/HA + BMP group. At 6 weeks, CaS/HA + BMP + ZA-l group had the highest BV among all groups. The CaS/HA + BMP group was still higher than CaS/HA and lower than CaS/HA + BMP + ZA-s group and CaS/HA + BMP + ZA-l group. The BMD of CaS/HA + BMP + ZA-l group was higher than all other treated groups at 6 weeks. Among the remaining five groups, the BMD of applied ZA (CaS/HA + ZA-s, CaS/HA + ZA-l, CaS/HA + BMP + ZA-s) was higher than without ZA application (CaS/HA, CaS/HA + BMP) (Fig. 3, Fig. S2). The quantification results of the ROI in this study compared with the same ROI in the SHAM group of one of our unpublished experiments and comparisons from 3 to 6 weeks are shown in Fig. S3.

### 2.4 Histological staining: CaS/HA local co-delivery of rhBMP-2 and ZA enhances bone formation

**Fig. 4:**
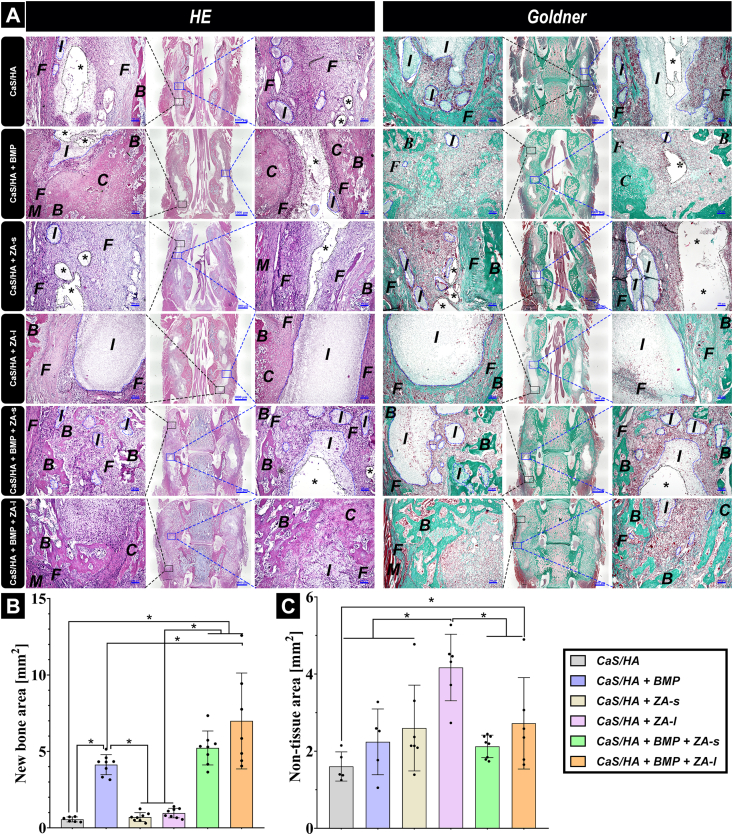
**(A)**, Representative histological images of the surgical lumbar fusion site in all treated groups at 3 weeks after surgery stained with HE and Goldner's trichrome and the corresponding quantification of **(B)** new bone area and **(C)** non-tissue area (including areas of remaining biomaterial and cavities). I: implant (blue dotted lines: residual, unresorbed material), B: new bone, C: cartilage, F: fibrous tissue, M: muscle tissue, stars (*) indicate cavities (black dotted lines). The black and blue rectangle areas of the overview images indicate the end (left high magnification images) and middle (right high magnification images) regions of the scaffolds, respectively. Overview images scale bars = 1000 μm and high magnification scale bars = 100 μm. Data are presented as means ± SD. *p < 0.05.

At 3 weeks hematoxylin and eosin (H&E) as well as Goldner's trichrome histological staining were performed. The results showed that the biomaterial scaffolds were surrounded by fibrous tissue and were infiltrated by bone marrow-like tissue, but no connective tissue ingrowth could be detected in the CaS/HA, CaS/HA + ZA-s and CaS/HA + ZA-l groups. The granular cells were well infiltrated within the biomaterial scaffold, while the undegraded biomaterial remains were not obvious and large cavities were visible in the CaS/HA and CaS/HA + ZA-s groups. Combined with μCT and the following fluorescent staining results, cavities were formed due to their loose structural assembly being removed during sample processing. The boundary between the scaffold and fibrous tissue was clear in the CaS/HA + ZA-l group because the dense biomaterial residues were not easily removed during the specimen preparation process. In the rhBMP-2-containing groups, many new bone trabeculae formed into the scaffold. Since there was a lot of cartilage tissue in the new bone tissue, it seems likely that new bone was formed through endochondral osteogenesis. Trabecular islets were filled with CaS/HA remnants or granular cells and the boundary between new bone and surrounding paraspinal muscle was clear. Eventually, the adjacent transverse processes were joined by newly formed bone resulting in PLF of lumbar. In the CaS/HA + BMP and CaS/HA + BMP + ZA-s groups, the undegraded biomaterial remains were not obviously visible and large cavities were observed in the center of the new formed tissue. This may result from the rhBMP-2-mediated resorption activity in combination with fluorescent staining and μCT results. In the CaS/HA + BMP + ZA-l group, the undegraded scaffold remains were still obvious, but the scaffold degraded significantly faster compared with the CaS/HA + ZA-l group. One reason could be that the inhibition of biomaterial degradation by local ZA was partial counteracted by rhBMP-2-mediated cellular effects, while local introduction of ZA into the scaffold does not impair rhBMP-2-induced bone formation in the scaffold (Fig. 4A). Semi-quantitative analysis of the new bone at 3 weeks further determined that groups containing rhBMP-2 showed much more new bone formation compared with rhBMP-2-free groups. Additionally, the area of newly formed bone in the CaS/HA + BMP + ZA-l group was larger than that of CaS/HA + BMP and CaS/HA + BMP + ZA-s groups, but the differences between the CaS/HA + BMP + ZA-l and CaS/HA + BMP + ZA-s groups, and between the CaS/HA + BMP + ZA-s and CaS/HA + BMP groups were not statistically significant (Fig. 4B). Loose remnants of the biomaterial were removed during the specimen preparation process, thereby the quantification of non-tissue area (including areas of remaining biomaterial and cavities) was performed to roughly quantify the remaining non-resorbed biomaterial in different treatment groups. The results showed that local delivery of ZA significantly slowed down the resorption of the scaffold but did not hinder the effects of rhBMP-2. Notably, the CaS/HA group exhibited lower values due to removal of biomaterial during the specimen preparation process, which resulted in the surgical lacking support structures, thereby reducing the quantified area (Fig. 4C).

### 2.5 Fluorescent staining: local co-delivery of rhBMP-2 and ZA provides spatiotemporal effects on bone formation

**Fig. 5:**
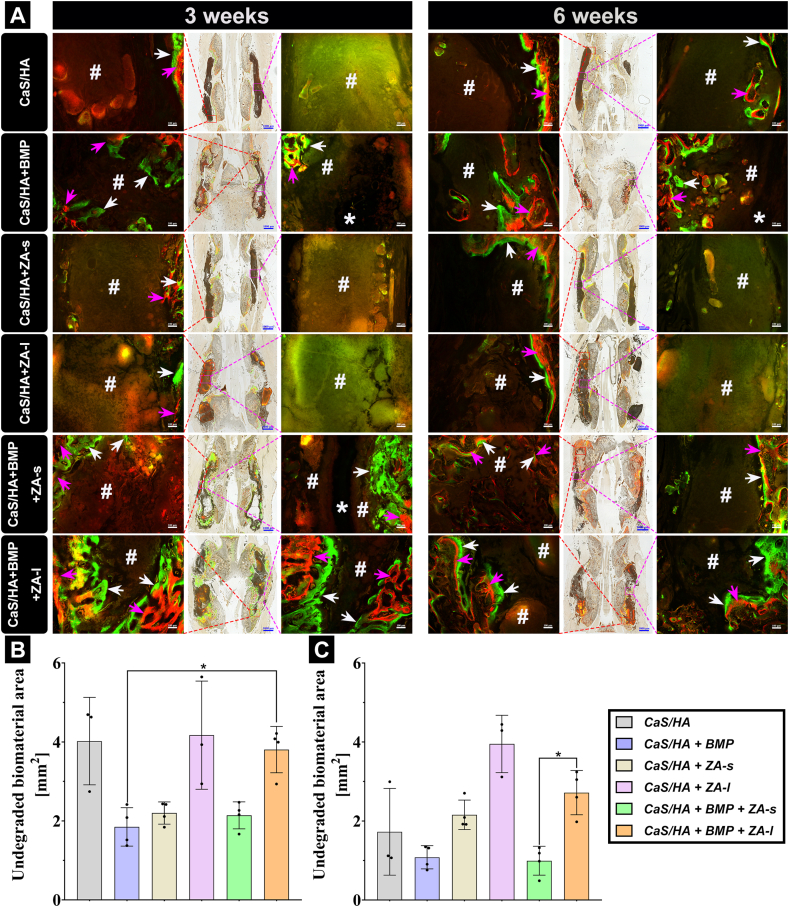
**(A),** Representative histological images of Alizarin Red/Calcein fluorescent labeling *in vivo* analyzed with fluorescence microscopy and the corresponding quantification of the unresorbed biomaterial area at **(B)** 3 and **(C)** 6 weeks based on fluorescent staining images. Purple and white arrows indicate red (Alizarin Red fluorescent dye labelling) and green (Calcein fluorescent dye labelling) new bone mineral deposition bands, respectively. “#” and “*” indicate residual, unresorbed material and formed cavities, respectively. The red and purple rectangles of the overview images indicate the end (left) and middle (right) regions of the scaffolds, respectively (both high magnification images). Overview images scale bars = 1000 μm, magnified images scale bars = 100 μm. Data are presented as means ± SD. *p < 0.05.

Alizarin red (red band) and calcein (green band) labeled newly mineralized bone *in vivo* at 7 and 3 days before euthanasia, respectively. These double label colors in the merged images were mixed in some areas, indicating that deformation/remodeling of new bone was constantly taking place during bone formation [30]. The double-labeled bone mineralization bands in each group were narrowed from 3 to 6 weeks, indicating that regardless of the strength of osteogenesis activity, the bone formation activity at 6 weeks was weakened. Specifically, bone formation was the same as observed with histological sections, and there was no bone formation activity (purple rectangle) in the scaffold of the biomaterial group without rhBMP-2. Here, bone formation only occurred in the transverse process or lamina and was more likely to occur in the decortical surgical area (red rectangle). New bone formation and mineralization occurred throughout the entire scaffold in the rhBMP-2-containing scaffold groups, connecting the transverse processes to promote spinal fusion. Surprisingly, CaS/HA + BMP + ZA-l had wider bands of mineralized new bone at both time points, indicating a more persistent bone formation activity than CaS/HA + BMP and CaS/HA + BMP + ZA-s group. The red-marked mineralized bone band was located at the periphery of the scaffold whereas the green one was located at the central side. This indicates that new bone grew from the periphery to the center of the scaffold based on the fact that recruitment of stem cells from the surrounding tissues is necessary to promote the bone formation process. Red/green-mixed bone deformation/remodeling areas can be seen in the areas of new bone in all three rhBMP-2-containing groups. Compared with CaS/HA + BMP + ZA-s and CaS/HA + BMP + ZA-l, staining in the CaS/HA + BMP group was more obvious at 3 weeks, indicating that the addition of ZA slowed down the bone remodeling process. In addition, large cysts were seen in newly formed bone areas in the CaS/HA + BMP and CaS/HA + BMP + ZA-s groups, but not in the CaS/HA + BMP + ZA-l group as well as in the other groups. The results also showed that the biomaterial degradation rate of CaS/HA + ZA-l and CaS/HA + BMP + ZA-l group was slower than in the other treated groups, in which CaS/HA + BMP + ZA-l group degraded faster than CaS/HA + ZA-l group (Fig. 5A, Fig. S4). The quantitative analysis of the remaining unresorbed scaffolds revealed that incorporating ZA indeed reduced the scaffold degradation rate without compromising the effectiveness of rhBMP-2. Over the period from 3 to 6 weeks, significant degradation of scaffolds was observed in all groups except for the CaS/HA + ZA-l group. This underscores the remarkable biological targeting effect of locally loaded ZA via CaS/HA carrier and its sustained release efficacy (Fig. 5B–C).

### 2.6 Osteoclast staining: local co-delivery of ZA inhibits pro-osteoclastic effect of rhBMP-2 during bone formation process

**Fig. 6:**
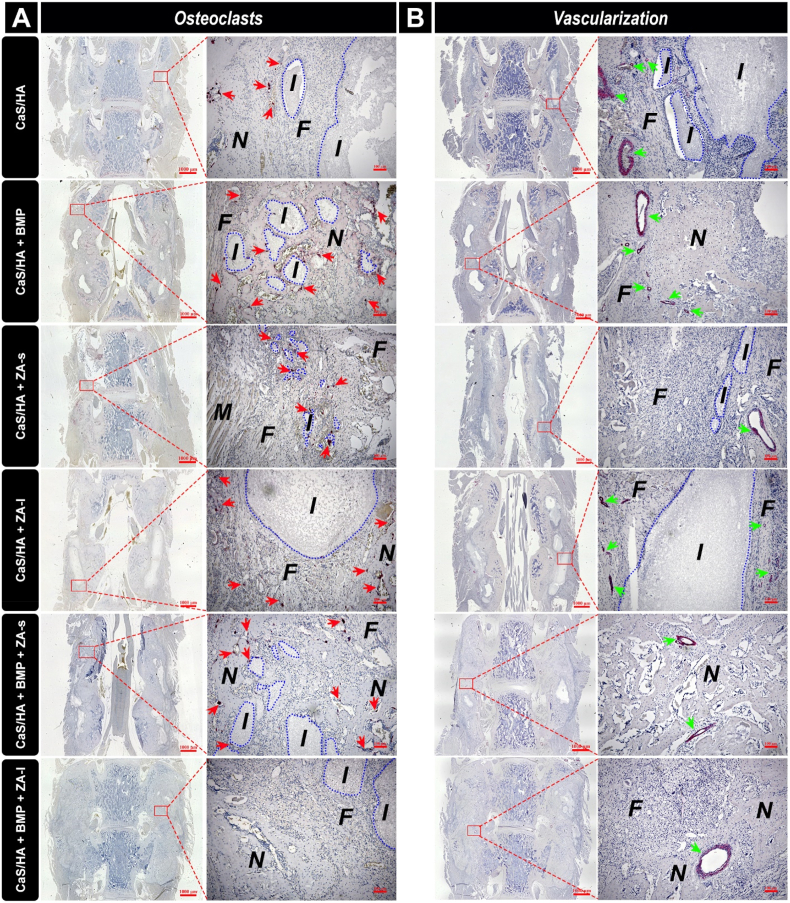
Representative histological sections stained for (**A**) tartrate-resistant-acid-phosphatase (TRAP, osteoclastic marker) and (**B**) α-SMA (blood vessels) in all treated groups at 3 weeks after surgery analyzed under a light microscope. Red arrows (left) and green arrows (right) indicate positive-stained osteoclasts and blood vessels, respectively. I: implant (blue dotted lines: residual, unabsorbed material), N: new bone, F: fibrous tissue, M: muscle tissue. Overview images scale bars = 1000 μm and magnified images scale bars = 100 μm.

The tartrate-resistant-acid-phosphatase (TRAP) staining results showed that the positive-stained osteoclasts were seen in the fibrous tissue surrounding the undegraded biomaterial residues in the CaS/HA, CaS/HA + ZA-s and CaS/HA + ZA-l groups, and no positive-stained cell was observed within the scaffold. These results indicated no obvious osteoclast activity inside the scaffold in rhBMP-2-free groups and local application of ZA mainly mediated the anti-osteoclast effect in the scaffold and affected the surrounding tissue of the scaffolds only in a weak manner. In CaS/HA + BMP and CaS/HA + BMP + ZA-s, many positive stained osteoclasts can be seen within the new bone tissue and surrounding fibrous tissue, but no positive-stained cell was observed within the new bone tissue of CaS/HA + BMP + ZA-l group (Fig. 6A).

### 2.7 Vascular staining: rhBMP-2 promotes ingrowth of blood vessels in new bone tissue

By α-smooth muscle actin (α-SMA) staining many blood vessels could be detected in the fibrous tissue and paraspinal muscle tissue surrounding the biomaterial residues in the CaS/HA, CaS/HA + ZA-s, and CaS/HA + ZA-l groups, whereas no blood vessels were observed within the scaffold. In the CaS/HA groups with rhBMP-2, blood vessels could also be observed among the new bone trabeculae within the scaffold. However, it was also observed that blood vessels were less distributed with in new bone tissue in the CaS/HA + BMP + ZA-l group than in the CaS/HA + BMP and CaS/HA + BMP + ZA-s groups in a limited number of specimens, which may be related to the denser residual biomaterials caused by the local delivery of ZA (Fig. 6B).

### 2.8 Bone biomarkers: local ZA application does not hinder systemic bone turnover

**Fig. 7:**
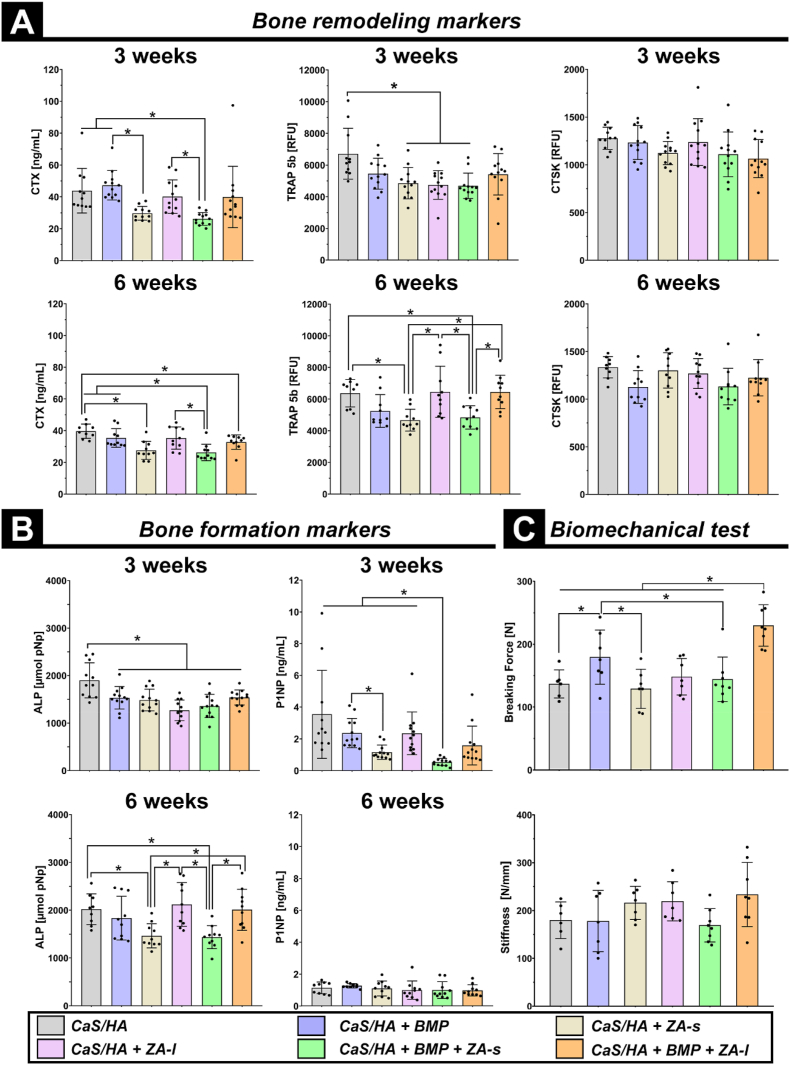
**(A**–**B).** Blood serum analysis regarding **(A)** CTX, CTSK and TRAP 5b (bone remodeling markers) and **(B)** ALP and P1NP (bone formation markers). **(C).** The breaking force and corresponding stiffness of the explanted lumbar at 6 weeks after surgery measured to assess the biomechanical properties of the spinal fusion effect in all treated groups. Data are presented as means ± SD. *p < 0.05.

Analysis on blood serum samples was used to evaluate bone turnover in which alkaline phosphatase (ALP) and N-terminal propeptide of type I procollagen (PINP) markers were used to assess bone formation [31]. Tartrate-resistant acid phosphatase 5b (TRAP 5b), C-terminal telopeptide of type I collagen (CTX), and Cathepsin K (CTSK) were used to assess bone remodeling [31]. Overall, the systemic administration of ZA, whether combined with rhBMP-2 or not, significantly hindered systemic bone remodeling as evidenced by lower blood levels of CTX at both 3 and 6 weeks and TRAP 5b at 6 weeks post-operation in CaS/HA + ZA-s and CaS/HA + BMP + ZA-s groups compared to other groups. In contrast, local application of ZA did not appear to hinder systemic bone remodeling, as demonstrated by no significant difference between CaS/HA vs. CaS/HA + ZA-l and CaS/HA + BMP vs. CaS/HA + BMP + ZA-l regarding CTX at 3 and 6 weeks and TRAP 5b at 6 weeks postoperatively (Fig. 7A). Similarly, this effect was also observed in bone formation markers displayed by ALP at 6 weeks and P1NP at 3 weeks (Fig. 7B). CTSK blood levels at both time points were not significantly different among all groups, which may be due to its hyposensitivity (Fig. 7A).

### 2.9 Biomechanical testing: CaS/HA co-delivery of rhBMP-2 and ZA significantly enhances lumbar biomechanics

In this study, the initial breaking force of samples was used to evaluate the PLF effect (Fig. S5). The breaking force in the CaS/HA + BMP group was significantly higher compared to the CaS/HA group and the biomechanical strength of PLF in the CaS/HA + BMP + ZA-l group was significantly higher than that of all other treated groups. Unexpectedly, the breaking force in the CaS/HA + BMP + ZA-s was lower than the CaS/HA + BMP group. The stiffness [32] in the CaS/HA + BMP + ZA-l group was higher than in all other groups, but this difference was not significant (Fig. 7C).

## 3 Discussion

The aim of the study was to investigate if a one-step surgical strategy to accelerate spinal fusion using a bioactive bone graft substitute could serve as an off-the-shelf alternative for bone transplantation. This goal was achieved by controlled co-delivery of two approved bone active molecules, rhBMP-2 and ZA, and an approved CaS/HA carrier. The bone forming effect of rhBMP-2 and the anti-resorptive effect of ZA were tested in a rat *in vivo* spinal fusion model. Increased net new bone formation as well as increased biomechanical strength in the newly formed bone between the adjacent transverse processes in the PLF was observed when both rhBMP-2 and ZA were co-delivered. Thus, CaS/HA-mediated controlled co-delivery of rhBMP-2 and ZA accelerated and lead to stronger spinal fusion and the regimen could act as an off-the-shelf bioactive bone substitute for PLF surgery.

### 3.1 Osteoconductive CaS/HA biomaterial does not promote PLF on its own

PLF is a clinically challenging task and has the highest failure rate among spinal fusion procedures [7]. There is no *in situ* native bone tissue between adjacent transverse processes and the span of adjacent lumbar transverse processes in the human is approximately 4.5 cm [3]. Therefore, bone grafts are necessary for PLF in humans where they act as an osteoinductive scaffold between the transverse processes. In rats, the distance between the L4-5 transverse processes is approximately 10 mm, a relatively large distance in a small animal and we believe the rat PLF model used in this study mimics the challenging spinal fusion situation in humans. Many regulatory approved and clinically used osteoconductive biomaterials allow new bone formation and ingrowth from underlying cancellous bone, but cellular recruitment and differentiation in an extra-osseous location such as in or on a muscle or on cortical bone remains a challenge [15,33]. In the present study, we found no new bone or vascular ingrowth in the osteoconductive CaS/HA biomaterial. Weak osseointegration between the scaffold and native bone was observed when CaS/HA only was used, suggesting that the osteoconductive CaS/HA scaffold only is not sufficient to promote PLF. The osteoconductive biomaterial needs recruitment and differentiation of mesenchymal stem cells into bone-forming cells, to induce new bone formation [15]. Therefore, functionalization of osteoconductive scaffolds with osteoinductive factors seems to be necessary for challenging extracortical PLF scenarios.

### 3.2 CaS/HA carrier-based delivery of rhBMP-2 promotes PLF

Laboratory-synthesized rhBMP-2 has been translated into bedside applications for a few decades and is considered the most potent osteogenic factor available currently. Despite the early success of rhBMP-2 usage in spinal fusion, more recent studies have indicated concerns regarding rhBMP-2 use. rhBMP-2 did not show advantages over iliac crest bone graft in spinal fusion, but its side effects have greatly limited its clinical application [9]. The ACS carrier co-packaged with rhBMP-2 in the Medtronic® product is considered to be suboptimal. The drawbacks associated with commercially ACS carrier delivered rhBMP-2 include low affinity for BMP and an early burst release. This results in a low retained dose of rhBMP-2, which is insufficient to meet the demands of new bone formation. Consequently, there is a necessity to increase the dose to achieve an adequate level of bone formation [3]. The ideal BMP carrier should have good biocompatibility, allow infiltration of blood vessels and cells, resist compression, and be molded to the contours of the bone [3]. Advantages of using a clinically-approved CaS/HA include high protein encapsulation, sustained drug release pattern, and improved surgical handling, which has been demonstrated in previous femur bone defect and ectopic muscular pouch experiments [15,26]. When rhBMP-2 was loaded into the scaffold, new bone formation occurred simultaneously along the periphery of the entire scaffold, indicating that osteogenic factors were uniformly distributed within the CaS/HA scaffold. Under the local cytokine and growth factor response to rhBMP-2 [34], good osseointegration between the CaS/HA scaffold and the transverse process was achieved. New bone successfully connected the transverse processes to achieve PLF and new blood vessels infiltrated the new bone tissue. Moreover, the newly formed intertransverse bony fusion was successfully transformed into biomechanical enhancement of the lumbar spine compared with the CaS/HA only group. This suggests that the osteoconductive CaS/HA scaffold functionalized with the osteoinductive factor BMP-2 and progenitor cells from the surrounding muscle and underlying cancellous bone promotes PLF. Previous studies suggested that large cystic lesions in the bone-forming zone and rapid degradation of biomaterials may be associated with BMP-induced strong osteoclast resorption activity [26,35], and a few osteoclasts could also be visualized in the bone-forming zone in the CaS/HA + BMP group of this study. CaS/HA is an optimal carrier for rhBMP-2 to promote spinal fusion, and the activation of osteoclasts seems not to be affected by the inorganic carrier material itself and rather related to the cytokine rhBMP-2.

### 3.3 Systemic and local delivery of ZA alone does not support PLF

Clinically, ZA is administered systemically for the treatment of diseases with increased bone turnover, such as Paget's disease, hypercalcemia of cancer-induced malignancies, and osteoporosis [19]. Many studies reported that systemic use of ZA accentuates stimulus of bone formation during fracture healing [36,37]. In a previous study, we found topical loading of ZA on the CaS/HA scaffold increased cancellous bone regeneration and improved bone implant anchoring [28]. But in this study, neither systemic nor local use of ZA was shown to have a positive effect on new bone formation or biomechanical enhancement. This may be related to applying the material onto the cortical bone of the transverse processes, which is an extra-osseous environment with no access to bone stimulating BMP, either from cancellous bone graft or exogenous BMP from the scaffold. Cortical and cancellous bone have been found to respond differently to ZA in our previous femoral defect-healing model study and we speculated that they require different healing stimuli during regeneration [26]. Therefore, application of ZA in combination with a CaS/HA biomaterial, but without added BMP was not sufficient for PLF either systemically or locally.

### 3.4 CaS/HA-based local ZA application with high target biological effects

Bisphosphonates have a long skeletal half-life and there is evidence that pamidronate can be found in urine samples up to 8 years after systemic administration in children [38]. Persistent low bone remodeling after discontinuation of bisphosphonates interferes with normal bone formation and repair during growth in children [[39], [40], [41]]. Bisphosphonates cross the placenta and a drug that remains in bone tissue in adulthood may affect fetal development during pregnancy. Skeletal abnormalities have been observed in the offspring in animal models [40,41]. In humans, metaphyseal “zebra lines” were observed on radiographs in a case report of a teenage boy treated with intravenous ZA for *osteogenesis imperfecta* [42]. In this study, systemic administration of ZA suppressed blood levels of the bone remodeling markers CTX and TRAP and the bone formation markers ALP and P1NP, whether or not combined with rhBMP-2. Therefore, the safety of systemic administration of bisphosphonates is a concern, in addition to common side effects that occur initially after the drug is administered. Local *in situ* administration of bisphosphonates shows high bioavailability in targeted areas [22] and this approach may bypass the side effects of systemic ZA, improving its safety profile in spinal fusion applications. A promising finding was that ZA delivered locally by CaS/HA mainly played a therapeutic role in sustained release inside the scaffold with only a weak effect on the surrounding tissues. Blood levels of bone biomarkers were not affected by local ZA application compared to the corresponding systemic ZA application group. A recent study of our team further confirmed this conclusion [43]. By loading ^14^C-ZA into a micro- or nano-HA biomaterial, it was found that more than 99 % of ^14^C ZA still was locally retained within the defect and less than 0.1 % of the ZA could be detected in other organs, regardless of particle size, after 4 weeks in a rat tibial defect model. ZA is tightly linked and chemically bound to the HA phase in a biphasic scaffold [15,43]. The targeted high biological effect of ZA significantly reduced the degradation of CaS/HA. The degradation process of a ceramic biomaterial is thus regulated by two mechanisms, 1) dissolution due to the inherent solubility of the biomaterials CaS phase, and 2) cellular degradation of the HA by osteoclasts, known as resorption [44]. Compared with the CaS/HA only group, systemically-administered ZA had no significant effect on new bone formation and biomaterial degradation. This further confirms that the local application of ZA has higher bioavailability than systemic administration in the targeted area, while the lower biological effect of systemic ZA in the target area may be due to a dilution effect by the systemic distribution, the cortical bone-dominated transverse process, or the poor blood supply in the inter-transverse process.

### 3.5 Systemic ZA does not significantly enhance rhBMP-2-promoted PLF efficacy

ZA induces osteoclast apoptosis through the mevalonic acid pathway [18,19]. ZA appears to be beneficial to the net bone formation by inhibiting or delaying the osteoclast effect caused by the added rhBMP-2. In previous studies, systemic ZA therapy was combined with BMPs to achieve dense and robust fracture unions [45,46] and facilitate spinal fusion [18] by inhibiting the BMP osteoclast effect. In this study, systemic administration of ZA combined with rhBMP-2 also gained higher BV and BMD than rhBMP-2 administration alone at both time points. The BMD of new bone was also higher with local ZA combined with rhBMP-2 at 3 weeks. This may be due to a relatively weaker anti-osteoclastic activity and earlier bone remodeling due to the lower targeted biological effect of systemic ZA. More osteoclasts in the new bone tissue were visible in the CaS/HA + BMP + ZA-s group than CaS/HA + BMP + ZA-l group, which further proves that its reduction of the rhBMP-2-induced osteoclast effect was weaker with systemic than with local administration. The weak anti-osteoclast effect also resulted in the inability to prevent BMP-induced bone cyst formation and slow down the degradation rate of the CaS/HA carrier. The scaffold's relatively fast degradation led to the quick release of rhBMP-2 from the CaS/HA biomaterial. This rapid release may explain the weaker bone formation activity observed at 6 weeks compared to the CaS/HA + BMP + ZA-l group, which had the sustained release of rhBMP-2. Additionally, the results of the three-point bending test showed no significant lumbar biomechanical enhancement, further indicating the fragility of the newly formed bone tissue in the CaS/HA + BMP + ZA-s group.

### 3.6 CaS/HA co-delivery of rhBMP-2 and ZA synergistically promotes PLF

Based on the high target biological effects of local delivery of ZA using a CaS/HA carrier, local co-delivery of ZA and rhBMP-2 may inhibit the pro-osteoclast activity of rhBMP-2 to obtain more net new bone formation. Furthermore, the *in-situ* effect of local ZA application may avoid the side effects of the systemic use of ZA for spinal fusion. The FDA-approved ACS carrier for rhBMP-2 is suboptimal for delivery of ZA due to a lack of ZA-binding domains such as in the case of HA [47]. An *in vivo* muscle pocket experiment showed that an ACS scaffold co-delivering rhBMP-2 and ZA had a significantly lower bone formation capacity compared to a gelatin-hydroxyapatite-calcium sulfate scaffold [48]. Based on this, the resorbable biphasic CaS/HA carrier has significant advantages of co-delivering rhBMP-2 and ZA. The results above indicated that in addition to encapsulation of rhBMP-2 into the CaS/HA biomaterial, the HA phase of the biomaterial had high accretion for ZA, in which the added ZA mainly acts inside the scaffold. Previous studies showed that the CaS phase of CaS/HA biphasic carrier encapsulates rhBMP-2 by physical entrapment while preserving the biological activity of the protein [15]. In contrast, ZA chemically binds to the HA phase [15,43]. CaS/HA co-delivery of rhBMP-2 and ZA achieved significant bone formation in both an *in vivo* muscle pouch model [15] and a femoral defect model [26], in which rhBMP-2 loading of CaS/HA biomaterials promoted bone regeneration. Local synergistic delivery of ZA helps to protect newly formed bone from early resorption caused by the addition of rhBMP-2 to activate osteoclasts. Bone formation is a complex process involving the coordination of various cascades and osteoclasts and osteoblasts are basically involved in the entire interaction process [44]. Osteoclasts also play a vital role in healthy bone remodeling [44]. The strong osteoclast inhibitory effect derived from the use of topical ZA may be criticized because it alters the physiological bone remodeling process. Bisphosphonates have been shown to directly enhance the proliferation, differentiation and bone forming activity of osteoblasts *in vitro*, and the osteoblasts can work independently in bone repair and reductions in osteoclast activity are expected to shift the balance between formation and resorption towards increased net bone formation [22]. In the fracture-healing model, it was found that the ZA used to inhibit osteoclasts does not affect the initial endochondral ossification stage. The inhibition of later bone resorption can eventually obtain more trabecular bone, which is beneficial to healing, and it is believed that bone remodeling is not necessary for initial fracture repair [36,37]. In the present study, the highly targeted biological effects of locally delivered ZA strongly inhibited the pro-osteoclastogenic activity of rhBMP-2, while the abundant cartilage tissue in the new formed bone indicated that ZA had no inhibitory effect on the initial stage of rhBMP-2 promoting new bone formation. Its anti-resorption ultimately resulted in more net vascularized new bone tissue, which has important implications for PLF procedures. The release profile of the local co-delivery regimen also avoids the effects of systemic ZA on remodeling of normal bone tissue elsewhere in the body. These results suggest that local co-delivery of ZA and rhBMP-2 based on CaS/HA carrier showed a synergistic effect, in which ZA does not hinder rhBMP-2 bone formation, and rhBMP-2 does not impair ZA inhibition of osteoclast biological effects.

### 3.7 CaS/HA co-delivery of rhBMP-2 and ZA spatiotemporally induces bone formation

The biological effects of both rhBMP-2 and ZA in our model are based on the degradation of biomaterial carriers to release the active factors [22,26]. The release kinetics of rhBMP-2 and ZA from CaS/HA biomaterials have been comprehensively characterized both *in vitro* and *in vivo* in our previous studies [15,28]. In the *in vitro* setting, 90 % of rhBMP-2 and 10 % of ZA were released within the initial first week [15,28]. Conversely, in the *in vivo* environment, the biomaterial exhibited a slower release of rhBMP-2 (15 %) and a faster release of ZA (16 %) in the first week, ultimately reaching approximately 57 % release of rhBMP-2 and 22 % release of ZA after a 4-week period [28]. This release profile was found to be superior to the release kinetics of rhBMP-2 from the current FDA-approved ACS carrier for rhBMP-2 delivery [49]. The biomaterial degradation rate in the CaS/HA co-delivered rhBMP-2 and ZA group was lower than that in the CaS/HA-loaded rhBMP-2 group, but the biomaterial residues were less than those in the ZA-only group. The synergistic effect of ZA and rhBMP-2 in biomaterial degradation is identical to its bone formation effect by coordinating osteoclast and osteoblast activities. The osteogenic differentiation of mesenchymal progenitor cells is initiated and maintained by rhBMP-2, physically encapsulated in the CaS phase and released over time [15,50]. The space from CaS degradation provides room for rhBMP-2-induced bone formation at the early stage. Concomitant with the degradation of the CaS phase, the CaS/HA scaffolds become porous over time, further synergizing through the dynamic structural remodeling of the material to enhance its osteoconductivity [15]. Due to the slow degradation of the HA phase of the scaffold, its slow degradation can provide space for bone formation and bone remodeling and avoid the lack of osteoconductive mediators in newly formed bone in later stage. Differential degradation rates between components of biphasic CaS/HA scaffolds and the application of scaffolds with bone active factors provide spatial effects for new bone formation. The local co-delivery of ZA to slow down the degradation of the scaffold can prolong the sustained release time and biological action time of rhBMP-2, thereby avoiding the disadvantage of premature burst release of rhBMP-2. A part of the loosely bound ZA embedded in the CaS phase of the CaS/HA biomaterial was released within 4 weeks [26], which may provide relatively strong inhibition of rhBMP-2 pro-osteoclast effect and slow down material degradation. The anti-catabolic effect of ZA combined with the anabolic effect of rhBMP-2 resulted in the CaS/HA + BMP + ZA-l group having the highest BV among all treatment groups at 3 weeks in this study. Predictably, ZA induced strong inhibition of osteoclasts resulted in low remodeling activity of rhBMP-2 induced new bone and relatively lower BMD of new bone. The large amount of new bone in the early stage can quickly connect the transverse process, which plays a pivotal role in PLF surgery that requires a large amount of bone tissue. Controlled sustained release of rhBMP-2 due to slow degradation of the scaffold under the action of ZA in the CaS/HA + BMP + ZA-l group allowed active bone formation to still be observed at 6 weeks, whereas the bone formation activity was very weak currently in other treatment groups. Based on massive new bone formation, the slow release of ZA tightly bound to HA provides mild anti-osteoclast effect to remodel new bone. This results in a higher BMD of the mass of new bone, promoting PLF and significantly improving its biomechanical stability. The synergistic effect of rhBMP-2 and ZA bioactive factors, combined with the CaS/HA biphasic scaffold and bone cells, offers differential effects of major early bone formation and later bone remodeling, make it particularly beneficial in the context of spinal fusion surgery scenarios.

### 3.8 CaS/HA controlled co-delivery of ZA potentially reduces rhBMP-2 dose for PLF

The current FDA-approved rhBMP-2 dosage is 1.5 mg/mL and is commercially marketed as a bone graft substitute by Medtronic® available in two different doses of 6 and 12 mg. Up to 40 mg of rhBMP-2 were used in ALIF for single-level degenerative lumbar disease in an investigational device exemption study [9,10,23]. The concentrations are extremely high compared to the <2 mg amount of BMP-2 found in the human body under physiological conditions [10]. Supraphysiological amounts of rhBMP-2 cause major side effects [9,10]. The previously reported dosage of rhBMP-2 for PLF in rats is 10 μg per animal, twice the dose in this study, including loading on ACS [34] or inorganic material carrier [51]. Directly comparing the doses used in this preclinical study to FDA-approved doses administered in clinical patients is challenging due to variations in biomaterial scaffold dimensions or bone graft volumes between preclinical animal experiments and clinical human procedures. By conducting an indirect concentration comparison, it was noted that the rhBMP-2 in the CaS/HA scaffold in this study was approximately 0.06 mg/cm^3^ [2.5 μg/(1.9 mm × 1.9 mm x 11.5 mm) rectangular scaffold], markedly lower than the rhBMP-2 dose delivered by ACS (1.5 mg/cm^3^), recognized as the most effective concentration for inducing new bone [9]. However, rhBMP-2 alone at a dose of 5 μg per rat demonstrated limitations in terms of biomechanical enhancement when delivered via CaS/HA. A higher rhBMP-2 dose may be required to obtain a superior therapeutic effect, which may be the reason for high doses of rhBMP-2 use in clinics. In fact, increasing the dose of BMP-2 only does not necessarily lead to higher fusion rates in spinal surgery [9] nor increased mechanical strength. The reduced efficacy associated with lower rhBMP-2 doses and the compromised safety profile of higher doses create a dilemma in obtaining an optimal dosing regimen [23]. When local ZA and rhBMP-2 were co-delivered, a substantial biomechanical enhancement of the lumbar fusion was obtained compared to rhBMP-2 alone. This suggests that the anabolic and anti-catabolic coupling effects of rhBMP-2 and ZA in spinal fusion surgery may potentially allow a reduced dose of rhBMP-2, avoiding the side effects of rhBMP-2 while still achieving satisfactory fusion. However, it's crucial to clarify that the dosages or multiples mentioned are not an absolute or optimal recommendation. Furthermore, this also makes the application of rhBMP-2 combination with ZA more cost-effective in spinal fusion.

### 3.9 No heterotopic ossification in CaS/HA biomaterial loaded with rhBMP-2

Heterotopic ossification is the most recognized adverse event related to rhBMP-2 use [9]. Its formation is due to premature leakage of rhBMP-2 from the ACS carrier. The ACS is compliant to external forces and the drugs, if not bound, may be squeezed out of the collagen material by surrounding tissue compression [3,9]. It is also possible that deformation of the ACS carrier in the wet state leads to premature rhBMP release [48], although this allows it to fill cages of any geometry. CaS/HA biphasic material can form a microporous osteoconductive matrix for clinical use as a bone void filler after the solid phase and liquid phase have been mixed [26]. The construct, has stronger mechanical properties than ACS - at least initially. The injectability allows it to be molded into any geometric shape as it sets *in situ*. There was no heterotopic ossification in any of the rhBMP-2 containing samples in this study. This indicated that the CaS/HA carrier did encapsulate the rhBMP-2 well. A previous *in vitro* study showed that co-delivery of rhBMP-2 and ZA had no significant effect on the stiffness of CaS/HA scaffold compared with containing rhBMP-2 or pure CaS/HA scaffolds [15]. However, this study showed that containing ZA scaffolds with or without rhBMP-2 (CaS/HA + ZA-l, CaS/HA + BMP + ZA-l group) became brittle and partially fractured during the implantation process. This may be due to the separation of the CaS and HA phases after ZA chemically binding HA, or the addition of ZA containing a large amount of solvent, which requires further exploration. Histology showed that only fibrous tissue grew into the fracture site, but no bone tissue grew out of the scaffold, which indicated that the fractured scaffold did not affect the protein encapsulation effect. This will improve the feasibility and safety of this treatment regimen in future intervertebral fusion procedures. However, the application of this treatment option alone would be limited in the intervertebral fusion procedures and would need to be used in conjunction with a refillable cage.

## 4 Limitation

There are still some limitations in the current study. To assess clinically meaningful spinal stability at 6 weeks, the histological assessment had to forego, so the osteoclasts and vascularization in new bone tissue were not assessed in this study. To homogenize the assessment of bone formation in the surgical area, the ROI selection included part of the native transverse process bone tissue, rather than the evaluation of only new bone tissue. This study is limited to a preclinical PLF fusion experiment, and whether this treatment regimen has the same effect and safety in intervertebral fusion requires further large animal intervertebral fusion model experiments. The co-delivery of rhBMP-2 and ZA is superior to the delivery of rhBMP-2 alone in this study, but the lowest dose of rhBMP-2 synergistically acting with ZA and the optimal dose ratio between rhBMP-2 and ZA need to be determined by further studies.

## 5 Conclusions

This is the first study to investigate a CaS/HA biomaterial loaded with rhBMP-2 and ZA for local co-delivery in spinal fusion surgery. The CaS/HA scaffold alone or in combination with ZA either by systemic or local administration had no positive effect on spinal fusion. The CaS/HA containing rhBMP-2 showed good encapsulation, cellular and vascular infiltration within the biomaterial, and no heterotopic ossification. CaS/HA-loaded rhBMP-2 alone promoted new bone formation, but due to a pro-osteoclast effect resulted in decreased net bone synthesis due to bone resorption. The anabolic and anti-catabolic effects of rhBMP-2 and ZA in an optimal CaS/HA carrier can reduce the necessary dose of rhBMP-2 to obtain firm spinal fusion, which will potentially reduce the side effects of high doses and be more cost-effective. CaS/HA-loaded ZA would also bypass systemic inhibitory bone turnover effects, potentially raising concerns about its safety. The different degradation rates between the components of the biphasic CaS/HA scaffolds and the release of local bone active factors rhBMP-2 and ZA provided spatial effects for new bone formation to facilitate PLF and biomechanical strength. Since all components have been approved for clinical use, this treatment regimen will be more easily translated into clinical applications as a safe and cost-effectiveness alternative to autologous bone grafting in PLF.

## 6 Materials and methods

### 6.1 Preparation of CaS/HA biomaterial

The CaS/HA biomaterial (purchased from BONESUPPORT AB, Sweden) consists of a premixed powder of 60 wt% (wt%) CaS and 40 wt% HA. According to the manufacturer's protocol, the CaS/HA powder was mixed with the contrast agent iohexol, at a liquid/powder ratio of 0.43 mL/g, which allowed for 4 min for the slurry to cure during this process. In this study, the slurry was made in a 24-well plate for 30 s and then the CaS/HA slurry was transferred into cubic silicon molds (2 mm × 2 mm x 12 mm) with a spatula. The material slurry was required to be set for 30 min before taking the scaffolds out of the mold. Based on the experimental group, four different formulations of CaS/HA biomaterials were prepared. The first type was the pure CaS/HA scaffold which was prepared by mixing 1 g of CaS/HA powder and 430 μL of iohexol in a sterile 24-well plate. The slurry was transferred into the mold directly to obtain approximately 15 cubic grafts. Due to surface tension, the material was concaved at the top, which resulted in an approximately 1.9 mm × 1.9 mm x 11.5 mm (≈41.5 mm^3^) cuboid. The second type was the CaS/HA biomaterial containing rhBMP-2 (part of a InductOs bone graft kit, Medtronic, Dublin, Ireland). A total of 37.5 μg of rhBMP-2 was mixed with 430 μL of contrast agent to form the rhBMP-2 solution, which was mixed with 1 g of CaS/HA powder to achieve 15 cuboids of CaS/HA + rhBMP-2 containing 2.5 μg of rhBMP-2 per graft. The third type was the CaS/HA biomaterial containing ZA scaffold. Its procedure was the same as for the pure CaS/HA scaffold, except that the mixed solution contained 94 μL of ZA solution (4 mg/5 ml, Novartis, Basel, Switzerland) and 336 μL of iohexol. This liquid was mixed with 1 g CaS/HA powder to obtain 15 cubic CaS/HA + ZA biomaterial grafts, each cylinder containing approximately 5 μg ZA. The fourth type of scaffold was the CaS/HA biomaterial containing rhBMP-2 and ZA. A total of 37.5 μg of rhBMP-2 was mixed with 94 μL of ZA (0.8 mg/mL) and 336 μL of iohexol to form the rhBMP-2 and ZA solution, which was mixed with 1 g of CaS/HA powder to achieve 15 cylinders of CaS/HA + rhBMP-2 + ZA approximately containing 5 μg ZA and 2.5 μg rhBMP-2 per graft. CaS/HA slurry for approximately one graft volume was always wasted partly in the 24-well plate during transferring the contents into the silicone mold. Preparation of the biomaterial scaffolds was performed under sterile conditions in a laminar airflow bench and the grafts were implanted within 24 h of casting the biomaterial.

### 6.2 PLF model and surgery

132 male, 10 weeks old Wistar rats (weight: 390.5 ± 21.8 g, range: 346–476 g) were ordered from Janvier Labs (Le Genest-Saint-Isle, France) and housed at the Experimental Center of the Faculty of Medicine, Technische Universität Dresden. The animals were kept on a 12 h light-and-dark cycle and fed a standard diet with food and water *ad libitum*. Animals were anaesthetized by inhalation of a mixture of isoflurane/O_2_ (3 %) and maintained at a flow rate of 2 L/min. Then, animals were placed in prone position on a 37 °C warm heating pad, isoflurane was lowered to 2–2.5 %, which was maintained during the entire duration of the surgery. For pain management, meloxicam (2 mg/kg) was injected subcutaneously for analgesia before starting the surgery. The anesthetic depth was conformed by examining responses to stimuli, body tension, and physiological parameters. The low back of the animal was shaved and disinfected with iodophor and a sterile hole drape was placed at the surgical site before starting the procedure. A posterior midline incision was made along L4-L5 spinal process and the fascial incisions were made 2–3 cm on each side of the midline, and the transverse processes of L4 and L5 were exposed. The dorsal base of the L4 and L5 transverse processes were decorticated using a high-speed burr (Ø 2 mm), and the incision was rinsed thoroughly with saline to remove autologous bone debris. The scaffolds (CaS/HA, CaS/HA + ZA, CaS/HA + BMP-2, and CaS/HA + BMP-2 + ZA) were placed bilaterally touching the transverse processes of L4 and L5. Surrounding structures were guided back into their natural anatomic positions. The incision was closed in layers (muscles, fascia and skin) using absorbable sutures (Vicryl 4–0, Ethicon, Johnson & Johnson, New Brunswick, NJ, USA). After stopping the isoflurane flow, the animals were given 100 % oxygen until first sights of movement were visible. Then they were transferred back into their cage and allowed to wake up under a heating lamp while regularly monitored until full recovery. Animals were free to move after surgery. Since 5 μg ZA or 2.5 μg rhBMP-2 was contained in one scaffold, 10 μg ZA or 5 μg rhBMP-2 was finally administered topically in one animal. Systemic ZA administration was prepared from commercial vials in sterile saline and administered as a single subcutaneous injection of 0.1 mg/kg 1 week after surgery (Figs. S6–7).

### 6.3 Preparations for imaging, histology, and biomechanical testing

After an observation period of 3 and 6 weeks, animals were anaesthetized with isoflurane, followed by blood sample collecting via the intracardiac route, and then sacrificed by exposure to CO_2_ for euthanasia. All animals were subjected to X-ray before and after explantation of lumbar specimens following μCT measurement. At 3 weeks, two samples which were double labeled with alizarin red and calcein before sacrifice were used for fluorescence analyses. The remaining samples were used for histological hematoxylin-eosin (H&E), Goldner's trichrome, tartrate-resistant-acid-phosphatase (TRAP, osteoclasts), and immunohistochemical α-smooth muscle actin (α-SMA, vessels) staining. At 6 weeks, two samples labeled with fluorescent dyes were used for fluorescence analyses, the remaining samples were used for biomechanical testing.

### 6.4 X-ray photography

At the end of the respective observation period, all animals were radiographed after cardiac blood sample collection and sacrificing. Rats were placed in a prone position and radiographs of the entire lumbar spine were obtained using a microradiography device (MX-20, Faxitron, Tucson, USA) with 30-kV operating voltage and an exposure time of 5 s to obtain high-resolution. X-rays before explanation were primarily used to assess surgical lumbar segments and the presence of variants or associated complications to guide specimen collection and initially evaluate spinal fusion effect. After explanting the lumbar spine specimens, they were imaged again to evaluate spinal fusion outcome. Radiographic spinal fusion score was performed according to the classification system of Curylo et al. [29]. Since it is sometimes very difficult to fully differentiate CaS/HA scaffolds from new bone based only on X-ray images in the study, the score was performed in combination with the μCT scan results. The grade is 0–4, with 0 indicating no new bone formation and 4 indicating solid bone between transverse processes on both sides.

### 6.5 Micro-CT

After X-rays, the samples were placed in test tubes and scanned in a μCT instrument (vivaCT 40, Scanco Medical, Wangen-Brüttisellen, Switzerland) with following settings: x-ray energy 70 kVp, 114 μA, 8 W, voxel size 25 μm, integration time 200 ms. The most distal end of the L6 spinous process and the proximal end of L4 vertebral body were used to define the micro-CT scan area. Micro-CT image sets from each sample were reconstructed and analyzed using the Dragonfly software (ORS Inc., Montreal, Canada). A cuboid region of interest (ROI) of one side (width x height x length: 5 mm × 6.5 mm x 13 mm) was defined to quantify new bone formation. The details of ROI were described as follows: in coronal view, the medial side starts from the base of the L5 and L4 transverse processes, with a width of 5 mm outward, and the lower part started from the L5 transverse process with a 13 mm length; in axial view, starting from the base of the transverse processes and paralleled to the central axis of the vertebrae; in the sagittal view, starting from the base of L5 and L4 transverse processes, the height was 6.5 mm upward (Fig. S8). A threshold of 200 mg HA/cm^3^ was used to identify the bone and was kept constant across all samples. Bone volume (BV) which was measured in mm³ and bone mineral density (BMD) which was measured in mg HA/cm³ were used as two outcome variables for μCT assessment. In one animal, the sum of the BV of the left and right ROIs was taken as the final BV of the sample, and the average BMD of the left and right ROIs was taken as the final BMD of the sample.

### 6.6 Fluorescent staining for mineral apposition

The different fluorescent sequence labeling dyes were used to describe the time course of new bone formation *in vivo*. To gain a detailed understanding of the bone formation process, two rats in each group were subjected to serial fluorescent double-labeling at both time points to assess bone formation and mineralization *in vivo*. Alizarin red (3 wt % in 2 wt % NaHCO_3_, 30 mg/kg) was injected subcutaneously 7 days before euthanasia and calcein (2 wt % in 2 wt % NaHCO_3_, 20 mg/kg) 3 days before euthanasia, respectively. Animals were euthanized at the corresponding time points and lumbar spine specimens were removed, cut, embedded in Technovit 9100, grinded to a thickness of 50–100 μm, and imaged using a Keyence BIOREVO BZ-9000 microscope (Keyence, Osaka, Japan; alizarin red: tetramethyl rhodamine isothiocyanate filter, excitation: 545/25, emission: 605/70; calcein: green fluorescent protein filter, excitation: 470/40, emission: 525/50) [26]. The most representative stained section image from one sample was chosen for quantifying the area of unresorbed biomaterial using Image J Version 1.53e software (National Institutes of Health, Bethesda, MD, USA). Quantification was performed only on complete scaffolds obtained, and measurements of individual scaffolds were utilized for statistical analysis.

### 6.7 Histological evaluation of spinal fusion

Following micro-CT imaging, all samples were subjected to (immuno-)histological analysis at 3 weeks except for 2 randomly selected from each group for fluorescent staining. Specimens were decalcified with EDTA, embedded in paraffin, and serially sectioned at 2.5 μm thickness along the coronal plane parallel to the scaffold on both sides. The morphological analysis was evaluated based on H&E and Goldner's trichrome staining (Merck, Darmstadt, Germany) which were stained according to standard procedures. To evaluate the effect of drug-loaded scaffolds on bone formation, the most representative image of stained sections from one sample was selected and its newly formed bone area was qualified using Image J Version 1.53e software (National Institutes of Health, Bethesda, MD, USA). The average of left and right measurements of a sample when both sides were stained in most samples or one side measurement when only one side was stained in a few samples was used for statistical analysis. Additionally, non-tissue areas, including areas of undegraded biomaterials and cavities, were quantified by the same method. The activity of osteoclasts was analyzed by tartrate-resistant acid phosphatase staining (TRAP, Sigma-Aldrich, St. Louis, MO, USA). Vascularization of the spinal fusion area was analyzed based on α-smooth muscle actin staining (mouse anti-smooth muscle actin, clone 1A1, 1:750, Cat. #M0851, Agilent Dako, Santa Clara, CA, USA) following detection with the ImmPRESS-AP Anti-Mouse Immunoglobulin G Detection Kit (Vector Laboratories, Burlingame, USA) with Romulin (Biocare, Pacheco, USA) as chromogen. The evaluation of all histological sections was performed using a Keyence BIOREVO BZ-9000 microscope (Keyence, Osaka, Japan).

### 6.8 Biomechanical testing

After 6 weeks, all samples, except the 2 samples per group for fluorescent staining, were used for mechanical testing after μCT scan. During specimen preparation, the L4 and L5 vertebra were kept and the soft tissue attached to the specimen was carefully removed, and the scaffold and new bone tissue remained intact. All samples were thawed overnight at 4 °C the one day before testing and then held at room temperature 1 h before testing for defined conditioning. Harvested vertebra specimens were placed in a custom-made three-point bending jig within the testing machine (Z2.5 TH of ZwickRoell GmbH & Co. KG, Ulm, Germany) according to Fig. S5. The top indenter was connected to a 2.5 kN Xforce P load sensor of ZwickRoell. A pre-load of 10 N was applied to the specimens using a traverse speed of 0.5 mm/s. After a hold time of 10 s, load was applied to the specimens at a speed of 1 mm/s until the vertebral complete fracture and force, displacement and time data were recorded. The testing machine was controlled and the measurement data acquired using ZwickRoell's proprietary testxpert software. Accompanying measurements were performed with the ARAMIS SRX system (Carl Zeiss GOM Metrology GmbH, Braunschweig, Germany) for optical visualization of the load-dependent behavior. Based on the measurement images taken, analyses based on digital image correlation were performed on the full-field displacements of the spine specimens' surface, which allow an evaluation of the deformations. The obtained force-displacement curve and the initial measured peaking force during the test were used to evaluate the stiffness [32] and load capacity of the specimens, respectively. On this basis, the specimens with the different states of the vertebral destruction were analyzed in an objectively quantitatively measurable manner, and the initial breaking peak force of bone bridge or scaffold connecting transverse process fracture was used as a surrogate in terms of mechanical stability and strength of the PLF and provide perspectives on the achievable properties in newly formed bone (Fig. S9).

### 6.9 Serum analysis of blood samples

Blood was collected from all animals by the intracardiac route following centrifugation (2000 rpm, 10 min) to separate the serum from whole blood. Serum samples were stored until analysis at −80 °C. The day before testing samples were thawed overnight at 4 °C. The bone formation markers of procollagen type I N-terminal propeptide (P1NP) and alkaline phosphatase (ALP), and the bone resorption markers of C-terminal telopeptide of type I collagen (CTX), tartrate resistant acid phosphatase 5b (TRAP 5b) and cathepsin K (CTSK) were used to evaluate bone formation and bone remodeling, respectively [31]. While P1NP and CTX were measured using commercially available ELISA kits according to the manufacturer's protocol (P1NP: Rat/Mouse P1NP EIA, Cat. # AC-33F1; CTX: Rat-Laps CTX EIA, Cat. # AC-06F1; both from ids immunodiagnosticsystems, East Boldon, UK), ALP, TRAP 5b and CTSK were measured biochemically. For ALP, 20 μL of each serum sample were incubated with 100 μL 1 mg/mL p-nitrophenylphosphate in ALP buffer (0.1 M diethanolamine, 0.1 % Triton X-100, 1 mM MgCl_2_, pH 9.8) at 37 °C for 30 min. After stopping the reaction with 80 μL 1 M NaOH, optical density was measured at 405 nm and correlated to a calibration curve. For TRAP 5b, 10 μL of each serum sample were incubated with 50 μL 2.5 mM naphthol-ASBI-phosphate in TRAP buffer (0.1 M sodium acetate, 50 mM disodium tartrate, pH 6.1) at 37 °C for 30 min. The reaction was stopped by adding 125 μL 0.1 M NaOH following fluorescence intensity measurement (ex/em: 405/520 nm). For CTSK, 50 μL of each serum sample were incubated with 50 μL CTSK working solution (10 mM Z-LR-AMC in 0.1 M sodium acetate + 4 mM EDTA, pH 5.5) at 37 °C for 30 min. Afterwards, fluorescence intensity was measured at ex/em 365/440 nm. Optical as well as fluorescence intensity measurements were performed using a spectrophotometer (TECAN infinite 200 Pro, Tecan Trading AG, Switzerland).

### 6.10 Statistical methods

Data were analyzed using SPSS Statistics 20 statistical software (SPSS, Inc., Chicago, IL, USA) and GraphPad Prism 8.0.1 (244) Software (Inc., San Diego, USA). All data were tested for normality using the Kolmogorov-Smirnov test and for homogeneity of variances of normally distributed data using Levene's test. Differences between multiple groups were tested using one-way analysis of variance (ANOVA) with LSD's post hoc method in case of normally distributed and homogeneous variance data or using Welch's test with Tamhane T2's post hoc method in case of normally distributed and unequal variance data. The nonparametric Kruskal-Wallis test with Dunn's post hoc method was used to test non-normally distributed data. The two-way ANOVA with Sidak's post hoc test was used to test the difference between different times. All data are expressed as means ± SD and the level of significance was set at p < 0.05 (*).

### 6.11 Estimation of sample size and power

Group size and effect size has been considered for power calculation. The sample size in most reviewed studies concerning rhBMP-2 was 5–10 per group regardless of the animal species or model and there is a consensus in published work that the minimal number of animals per experimental group is four [3]. In addition, this study also references our previous studies involving ZA and ZA + BMPs in different bone healing models, showing that a sample size of 10 and higher for multiple treatment groups (>6) is suitable for statistical comparisons between multiple groups to draw concrete conclusions about the experimental outcome [26,45].

## Funding

L.L. thanks the Olav Thon Foundation (Grant Number: 21-90) for financial support. D.B.R. thanks Maggie-Stephens Foundation (Grant Number: 20202004), 10.13039/100002163Sten K Johnson Foundation (Grant Number: 2021–0592) and The Crafoord Foundation (2021–0550) for research grants.

## Institutional review board statement

The animal experiment was approved by the Local Animal Care Committee of Dresden University Hospital (protocol number: DD24-5131/354/10, 29 April 2016), and guidelines set by the National Institutes of Health for the use of experimental animals were followed.

## Informed consent statement

Not applicable.

## Data and materials availability

All data are available in the main text or the supplementary materials.

## CRediT authorship contribution statement

**Xinggui Tian:** Writing – review & editing, Writing – original draft, Visualization, Software, Methodology, Investigation, Formal analysis, Data curation, Conceptualization. **Corina Vater:** Writing – review & editing, Visualization, Project administration, Methodology, Investigation, Funding acquisition, Conceptualization. **Deepak Bushan Raina:** Writing – review & editing, Writing – original draft, Methodology, Investigation, Funding acquisition, Conceptualization. **Lisa Findeisen:** Methodology, Investigation. **Lucas-Maximilian Matuszewski:** Investigation. **Magnus Tägil:** Writing – review & editing, Funding acquisition, Conceptualization. **Lars Lidgren:** Writing – review & editing, Funding acquisition, Conceptualization. **Anja Winkler:** Writing – review & editing, Methodology, Investigation. **Robert Gottwald:** Writing – review & editing, Methodology, Investigation. **Niels Modler:** Writing – review & editing, Methodology, Investigation. **Klaus-Dieter Schaser:** Writing – review & editing, Funding acquisition. **Alexander C. Disch:** Writing – review & editing, Funding acquisition. **Stefan Zwingenberger:** Writing – review & editing, Supervision, Project administration, Methodology, Investigation, Funding acquisition, Conceptualization.

## Declaration of competing interest

The authors declare the following financial interests/personal relationships which may be considered as potential competing interests: L.L. is a board member of Ortho Cell, Australia. L.L., M.T., and D.B.R. hold stocks in Moroxite AB, Sweden. The authors declare that they have no other competing interests pertaining to this study.
