# Supplementary material for: Co-delivery of rhBMP-2 and zoledronic acid using calcium sulfate/hydroxyapatite carrier as a bioactive bone substitute to enhance and accelerate spinal fusion
Source: Bioact Mater. 2024 Mar 6;36:256–71. doi: 10.1016/j.bioactmat.2024.02.034 (PMC10937206; doi:10.1016/j.bioactmat.2024.02.034)
Supplement: Multimedia component 1 [file mmc1.docx]

**Supporting Information for**

**Co-delivery of rhBMP-2 and zoledronic acid using calcium sulfate/hydroxyapatite carrier as a bioactive bone substitute to enhance and accelerate spinal fusion**

**1. Supplementary Table**

**Table S1.** Study overview including treatment groups, sample size and evaluation techniques at 3 and 6 weeks.

**2. Supplementary Figures**

**Figure S1.** The X-ray images after transplantation of all treated groups at 3 weeks and 6 weeks.

**Figure S2.** The representative 3D reconstructions (lateral view) and two-dimensional (sagittal) slices obtained by μCT of harvested samples at 3 weeks and 6 weeks.

**Figure S3.** The quantification results of the ROI in this study compared with the same ROI in the SHAM group of one of our unpublished experiments and comparisons from 3 to 6 weeks.

**Figure S4.** The representative overview images of *in vivo* alizarin red/calcein fluorescent labeling merged red and green only under fluorescence microscopy.

**Figure S5.** The typical curve of three-point bending experiment and corresponding testing photographs in this study.

**Figure S6.** Surgical procedure used for creating the L4-5 posterolateral lumbar fusion in rats.

**Figure S7.** The rat PLF model operation time of all treated groups at 3 weeks and 6 weeks.

**Figure S8.** The region of interest (ROI) for μCT analysis to qualification the posterolateral lumbar fusion effect based on the μCT scan.

**Figure S9.** The testing procedure of three-point bending experiment to evaluate spinal fusion effect at 6 weeks post-operation.

Table S1. Study overview including treatment groups, sample size and evaluation techniques at 3 and 6 weeks.

| **Groups** | **Operated** | | **X-ray**  **(*After*)** | | **μCT** | | **Fluorescent staining** | | **Histology** | **Mechanical testing** | **Blood analysis** | |
| --- | --- | --- | --- | --- | --- | --- | --- | --- | --- | --- | --- | --- |
| **Time (Weeks)** | ***3*** | ***6*** | ***3*** | ***6*** | ***3*** | ***6*** | ***3*** | ***6*** | ***3*** | ***6*** | ***3*** | ***6*** |
| **CaS/HA** | 12 | 10 | 9 | 8 | 9 | 8 | 2 | 2 | 7 | 6 | 11 | 9 |
| **CaS/HA + BMP** | 12 | 10 | 10 | 9 | 10 | 9 | 2 | 2 | 8 | 7 | 12 | 10 |
| **CaS/HA + ZA-s** | 12 | 10 | 10 | 10 | 10 | 9 | 2 | 2 | 8 | 7 | 12 | 10 |
| **CaS/HA + ZA-l** | 12 | 10 | 10 | 10 | 10 | 10 | 2 | 2 | 8 | 7 | 12 | 10 |
| **CaS/HA + BMP + ZA-s** | 12 | 10 | 10 | 10 | 10 | 9 | 2 | 2 | 8 | 7 | 12 | 10 |
| **CaS/HA + BMP + ZA-l** | 12 | 10 | 10 | 10 | 10 | 10 | 2 | 2 | 8 | 7 | 12 | 10 |

**Notes:** Fluorescent staining and histology indicate alizarin red/calcein double-labeling staining *in vivo* and special histological staining *ex vivo*, respectively. "After" represents the X-ray detection after transplantation.

**Supplementary Figures**


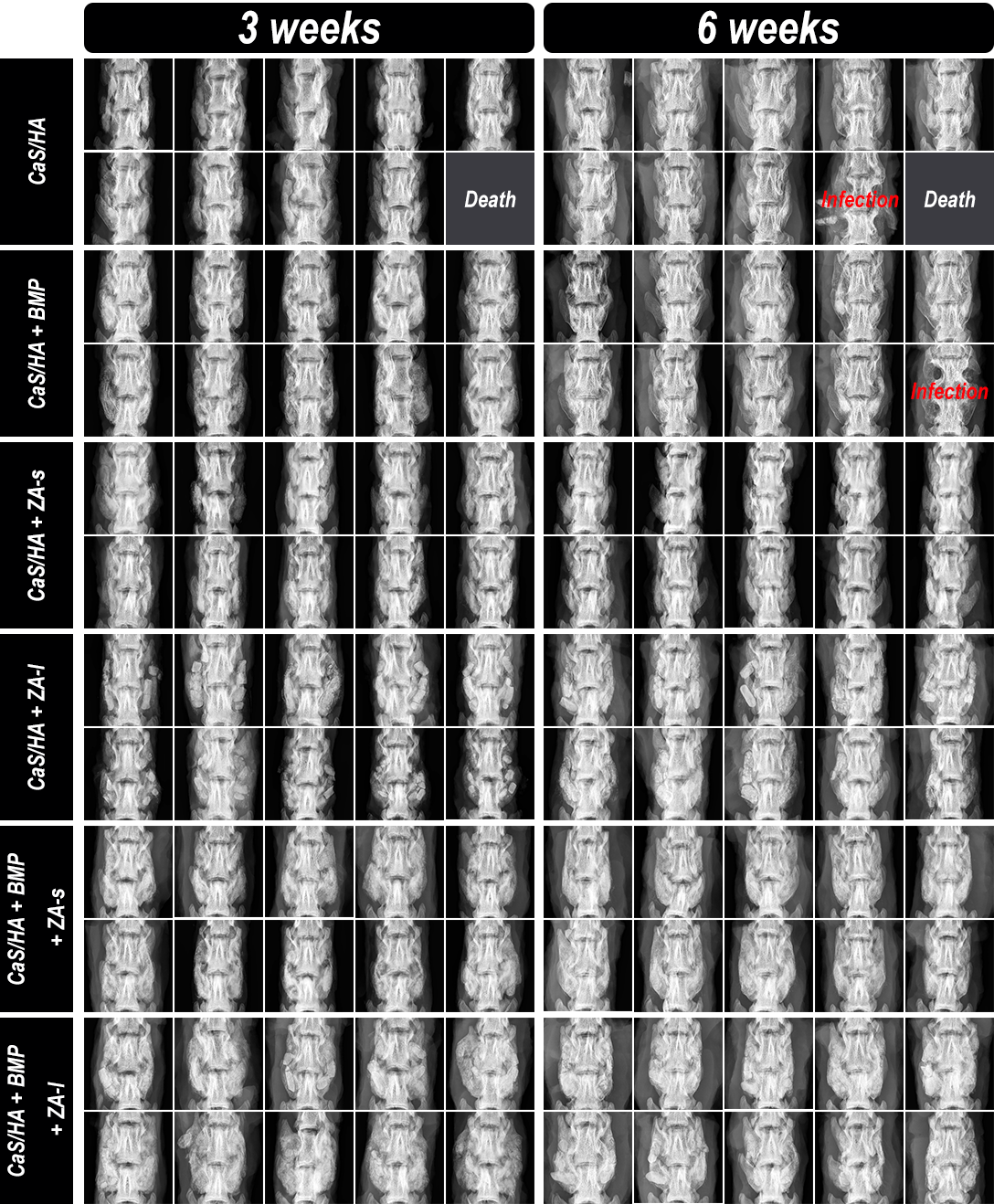


**Figure S1.** The X-ray images after transplantation of all treated groups at 3 weeks and 6 weeks. Two rats in the CaS/HA group (one from the 3 weeks observation time and one from the 6 weeks observation time) died during the observation time due to unknown reasons. 1 rat each in the CaS/HA group and the CaS/HA + BMP group were infected.


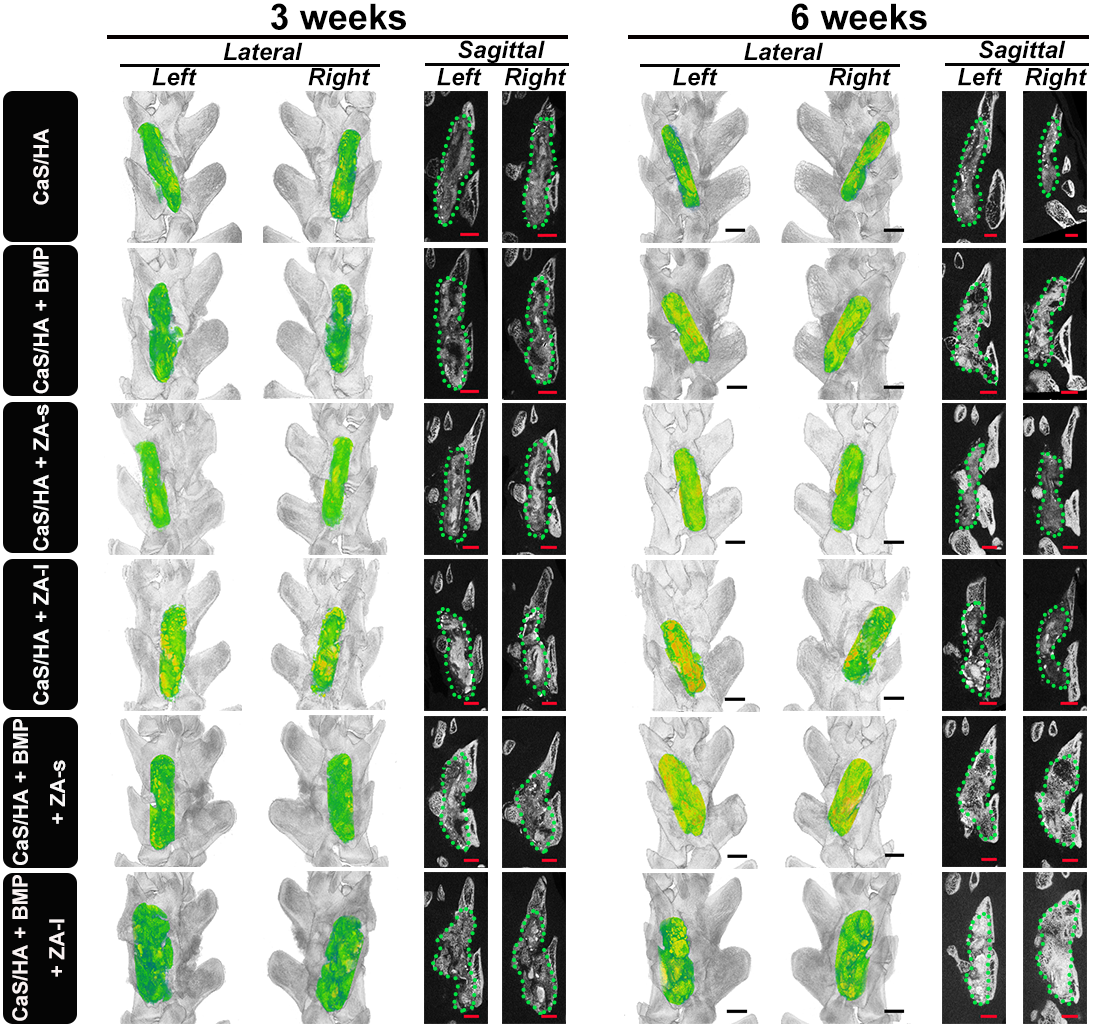


Figure S2. Representative 3D reconstruction images (lateral view) and corresponding two-dimensional (sagittal) slices obtained by μCT at 3 weeks and 6 weeks. The green rendered areas in µCT reconstruction images represent the implanted scaffold and new bone formation zones. Scale bars = 2 mm.


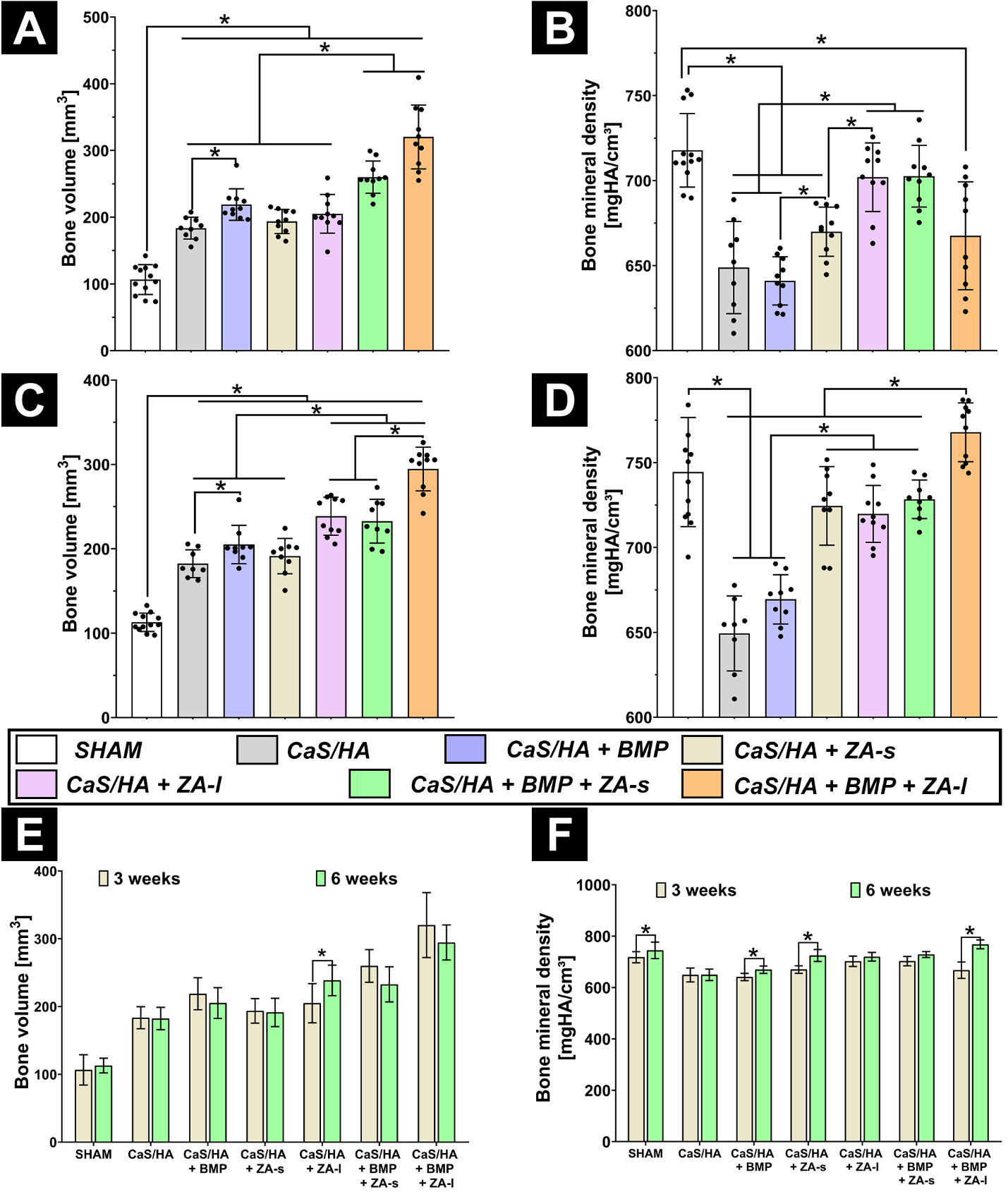


Figure S3. (A-D), The quantification results of the ROI in this study compared with the same ROI in the SHAM group of one of our unpublished experiments and comparison of (E) bone volume and (F) bone mineral density between 3 and 6 weeks in all treated groups. Data are presented as means ± SD. *p < 0.05.


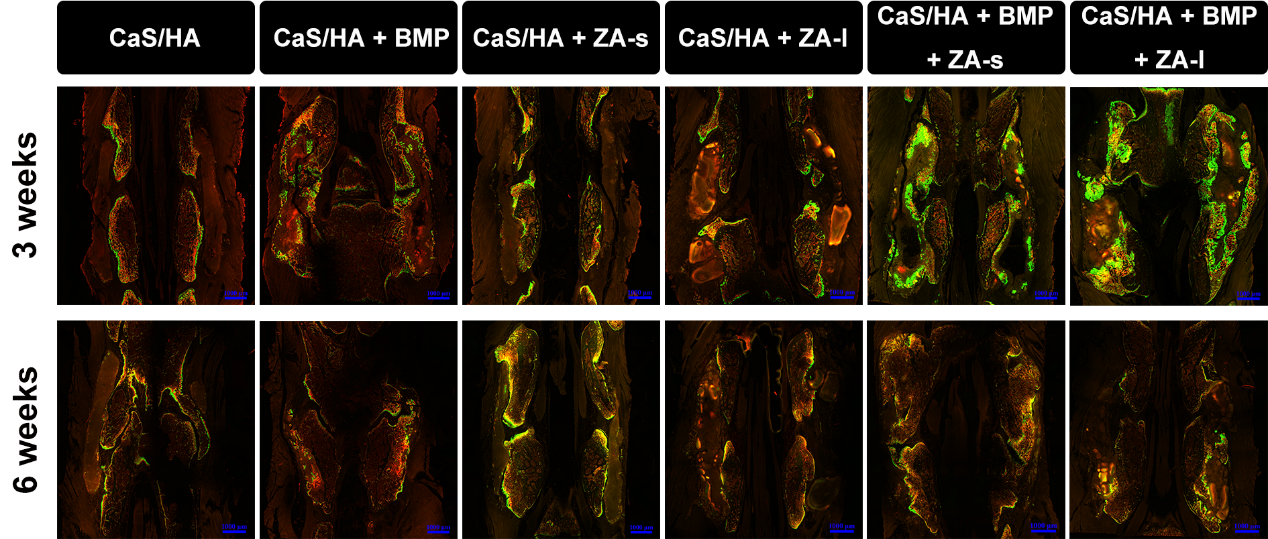


Figure S4. Representative overview images of alizarin red/calcein fluorescent labeling *in vivo* (merged red and green fluorescence). Scale bars = 1000 μm.


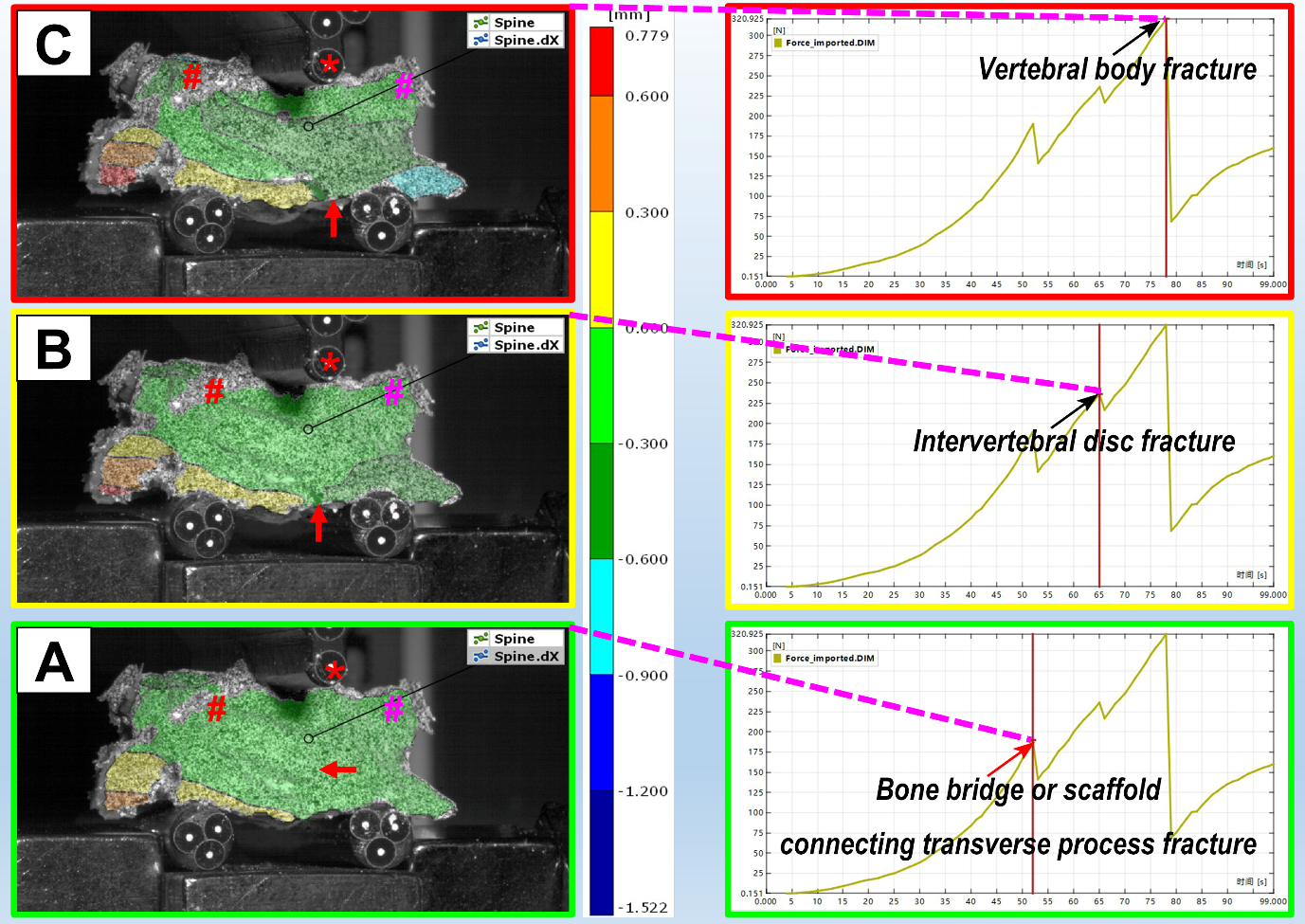


Figure S5. The typical load curve of three-point bending experiment (right curve charts) and corresponding images of specimen at defined load stages with overlaid result of displacement in spine-axial direction (left captured photos) were obtained from one sample in this study. The images represent the states: (A) the bone bridge or scaffold connecting transverse process fracture, (B) intervertebral disc fracture, and (C) the vertebral body fracture in individual specimen. The initial breaking force of the bone bridge or scaffold connecting transverse process fracture (A) is determined as a surrogate in terms of mechanical stability and strength of the posterolateral fusion effect in this study. The red “*” indicates the upper indenter. The purple “#” and red “#” indicate the L4 and L5 spinal process, respectively. The red arrows indicate the fracture locations of spine specimen.


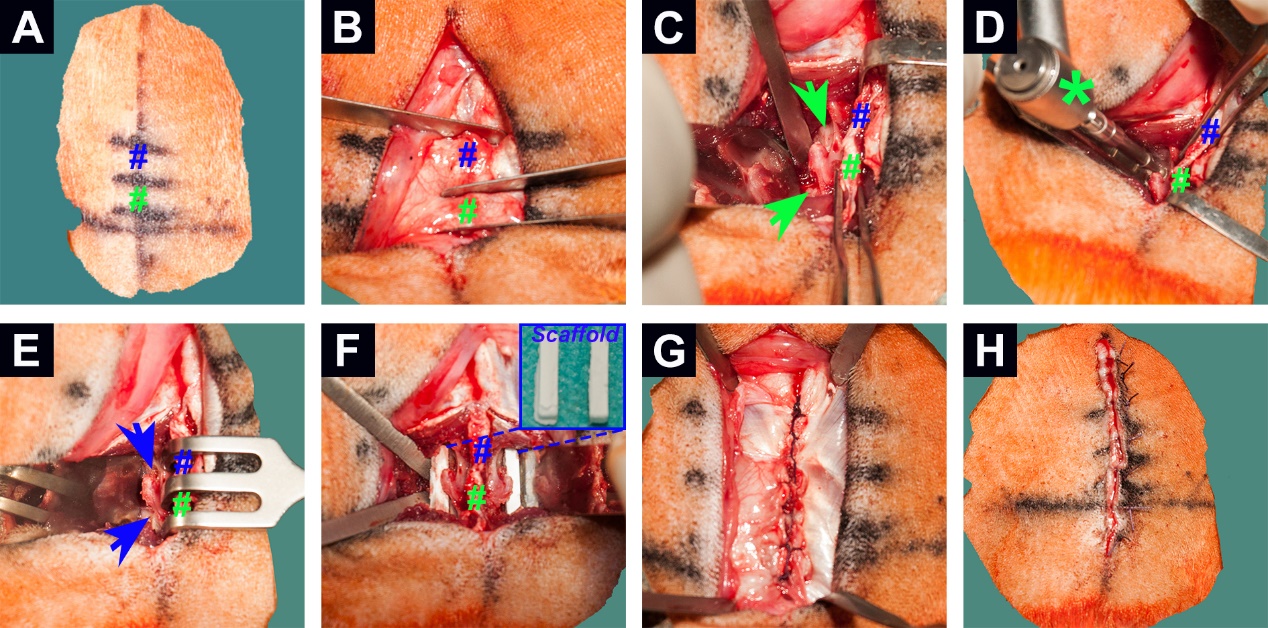


Figure S6. Surgical procedure of the L4-5 posterolateral fusion model in rats used in this study. (A) locate and disinfect the surgical site, (B-C) expose the surgical area of the L4-5 transverse process, (D-E) decorticalization of transverse process with a high-speed blur, (F) implant the corresponding scaffold according to the groups, (G-H) close the incision in layers (green * indicates high-speed blur, blue and green # indicate L4 and L5 spinal processes, green and blue arrows indicate transverse process and its corresponding decortical sites).


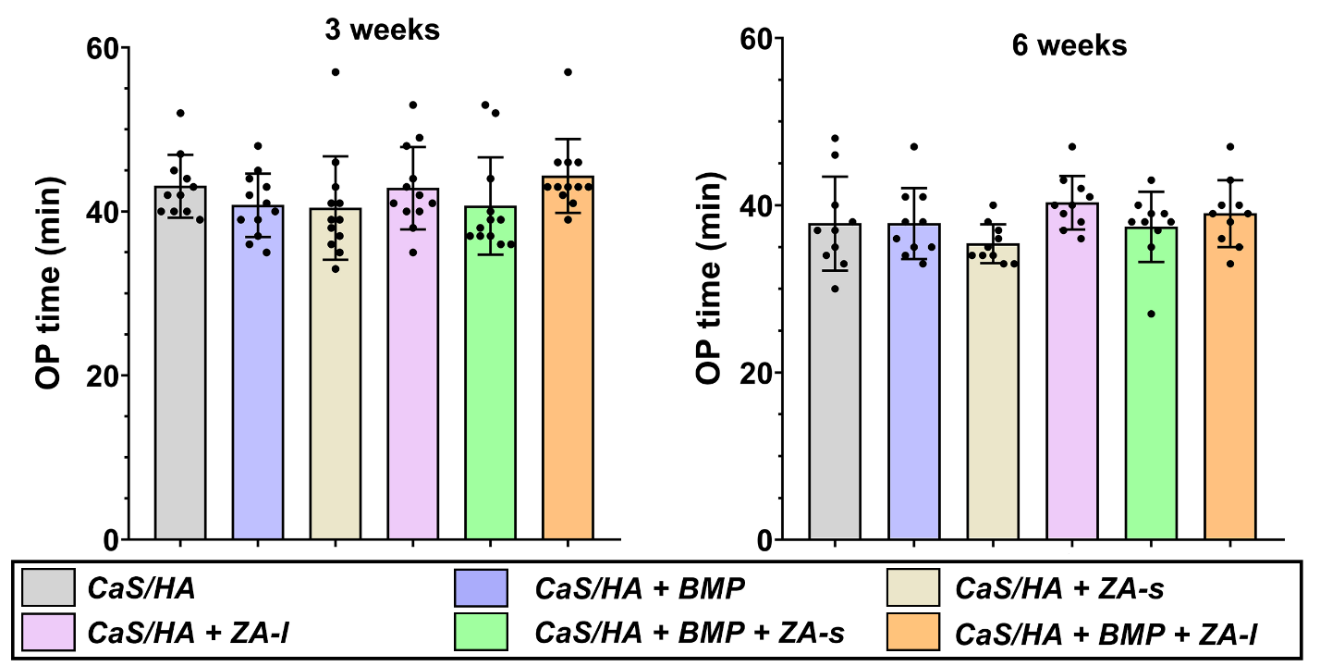


Figure S7. Operation time of posterolateral fusion model of rats in each treatment group used in this study at 3 and 6 weeks. Data are presented as means ± SD. *p < 0.05.


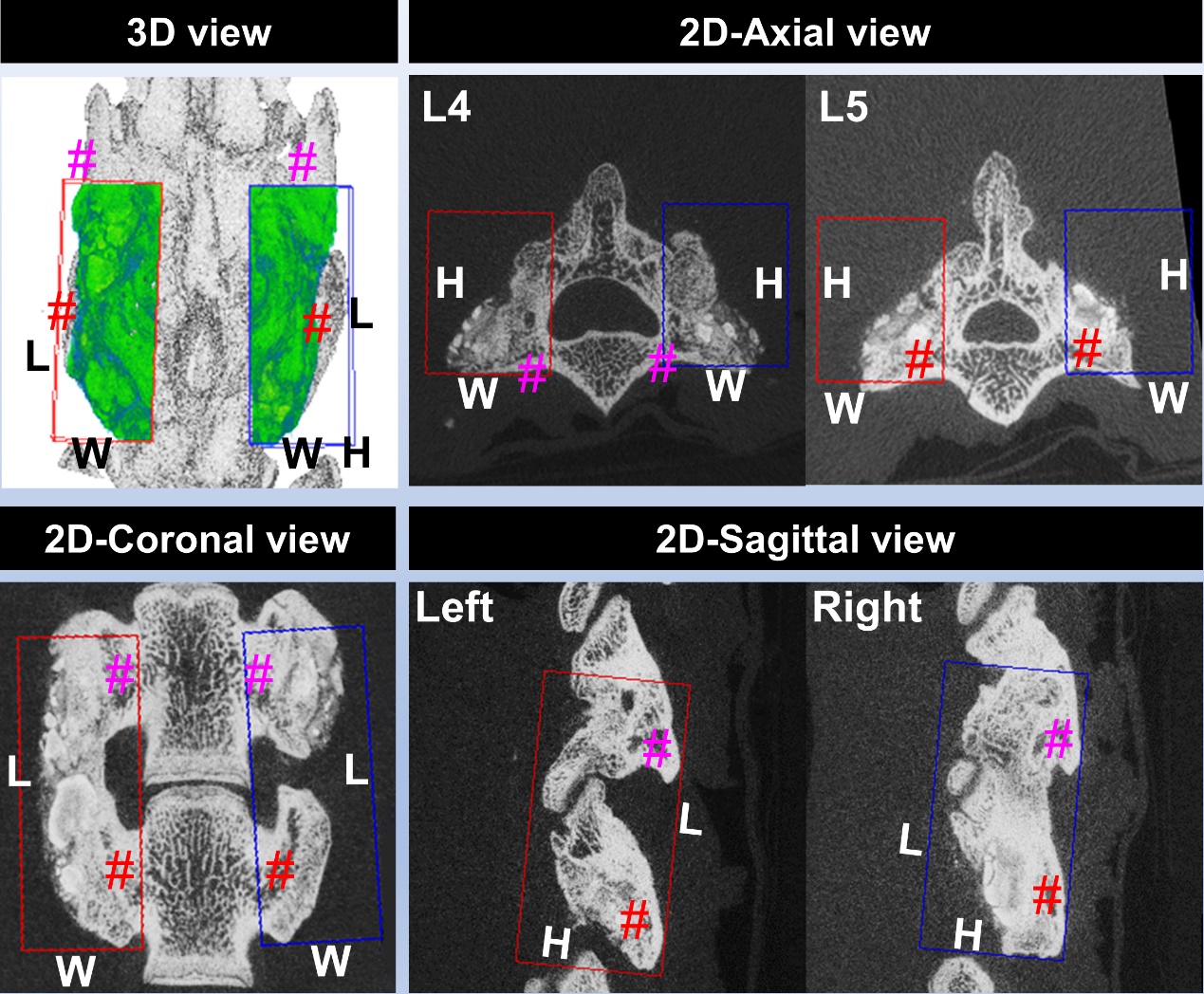


Figure S8. The region of interest (ROI) used for μCT analysis to quantify the posterolateral lumbar fusion effect based on the μCT scan in this study (purple and red # indicate L4 and L5 transverse processes; “L”, “W” and “H” text indicate length, width, and height of the cubic ROI).


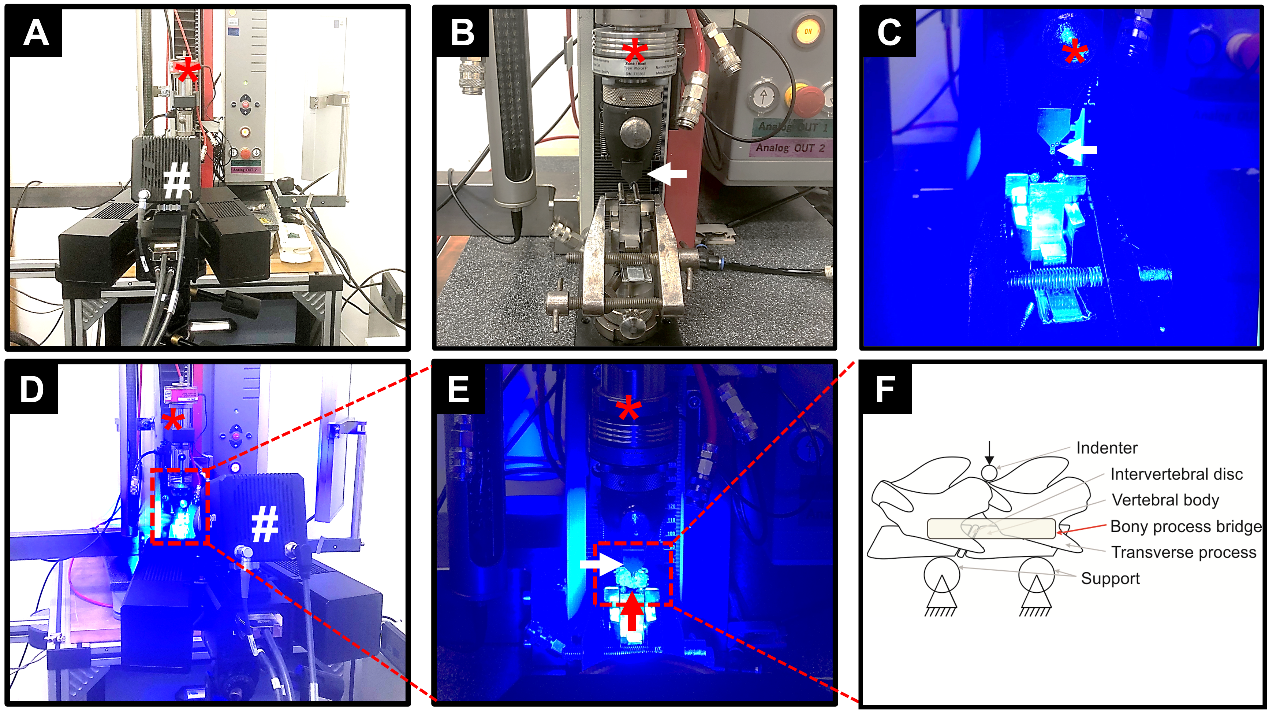


Figure S9. The testing procedure of three-point bending experiment used for evaluating biomechanical stability of spinal fusion at 6 weeks post-operation in this study. (A-C), the state before biomechanical testing; (D-E), the state during the biomechanical testing. (F), Schematic sketch of the biomechanical experimental setup. “*” and “#” markers indicate the testing machine and the sensor system, respectively. The white and red arrows indicate the upper indenter and the testing spine specimen, respectively.
